# Clinical Significance and Role of Lymphatic Vessel Invasion as a Major Prognostic Implication in Non-Small Cell Lung Cancer: A Meta-Analysis

**DOI:** 10.1371/journal.pone.0052704

**Published:** 2012-12-20

**Authors:** Jun Wang, Baocheng Wang, Weipeng Zhao, Yan Guo, Hong Chen, Huili Chu, Xiuju Liang, Jingwang Bi

**Affiliations:** 1 Department of Oncology, General Hospital, Jinan Command of the People’s Liberation Army, Jinan, China; 2 Tianjin Cancer Hospital and Institute, Tianjin Medical University, Tianjin, China; 3 Department of Outpatient, Military Command of Shandong Province, Jinan, China; 4 Department of Chinese Medicine, Affiliated Hospital of North Sichuan Medical College, Nanchong, China; Roswell Park Cancer Institute, United States of America

## Abstract

**Background:**

Lymphatic vessel invasion (LVI) exerts an important process in the progression and local spread of cancer cells. However, LVI as a prognostic factor for survival in non-small cell lung cancer (NSCLC) remains controversial.

**Methodology/Principal Findings:**

A meta-analysis of published studies from PubMed and EMBASE electronic databases was performed to quantity the effects of LVI on both relapse-free survival and overall survival for patients with NSCLC. Hazard ratios (HRs) with 95% confidence intervals (95% CIs) were used to assess the strength of these effects. This meta-analysis included 18,442 NSCLC patients from 53 eligible studies. LVI appeared in 32.1% (median; range, 2.8% to 70.9%) of tumor samples. In all, patients with LVI were 2.48 times more likely to relapse by univariate analysis (95% CI: 1.92–3.22) and 1.73 times by multivariate analysis (95% CI: 1.24–2.41) compared with those without LVI. For the analyses of LVI and overall survival, the pooled HR estimate was 1.97 (95% CI: 1.75–2.21) by univariate analysis and 1.59 (95% CI: 1.41–1.79) by multivariate analysis. Multivariate analysis showed a risk was 91% higher for recurrence (HR  = 1.91, 95% CI: 1.14–2.91) and 70% higher for mortality (HR = 1.70, 95% CI: 1.38–2.10) in LVI-positive I stage patients compared with LVI-negative I stage patients. Subgroup analyses showed similar significant adjusted risks for recurrence and death in adenocarcinomas, and a significant adjusted risk for death in studies that utilized elastic staining with or without immunohistochemistry in defining LVI.

**Conclusions/Significance:**

The present study indicates that LVI appears to be an independent poor prognosticator in surgically managed NSCLC. NSCLC patients with LVI would require a more aggressive treatment strategy after surgery. However, large, well-designed prospective studies with clinically relevant modeling and standard methodology to assess LVI are required to address some of these important issues.

## Introduction

Non-small cell lung cancer (NSCLC) accounts for approximately 80% of lung cancers and is the most common cause of cancer-related death worldwide [1]. Surgical resection is regarded as the current standard procedure for I-IIIA stage patients, but less than 15% of individuals diagnosed with NSCLC survive for 5 years. Especially in stage I patients, the 5-year survival rate after complete resection is reported to be 60 to 80%, suggesting that individuals who undergo surgery are a heterogeneous population and indicates the presence of occult metastasis at the time of surgical resection [2].

Prognostic factors may be useful for identifying subgroup of patients with a worse outcome and selecting a more aggressive treatment strategy such as adjuvant chemotherapy [3]. For example, the tumor-node-metastasis (TNM) staging system based on the characteristics of the tumor itself, regional lymph nodes, and potentially metastatic sites is an internationally accepted staging system. The seventh edition of the UICC/AJCC TNM staging system introduced in 2010 can be widely used to identify prognostic differences among patients with early-stage disease [2]. However, each patient’s prognosis varies significantly within each TNM stage, which makes it difficult to predict accurately the outcome for particular patient, especially for patients with early-stage lung cancer.

Pathological and biological factors involving in cancer development and progression, and genetic alterations have been identified to predict survival and improve treatment strategies of patients with NSCLC during the past decades [4]–[6]. Our previous meta-analysis concluded that the methylation of *RASSF1A* could serve as an independent prognostic marker for NSCLC [7]. Blood vessel invasion (BVI) also exerts an important influence on patient outcome. The relative risk of recurrence and death for an individual patient whose tumor showed BVI by tumor cells was nearly 4 and 2 times higher, respectively, than that of a patient whose tumor did not show BVI by tumor cells [8].

Lymphatic vessels are regarded as the important route by which neoplastic cells reach local lymph nodes [9]. Lymphatic vessel invasion (LVI) is made by detecting the tumor emboli within vascular channels lined by single layer of endothelial cells in the resected primary tumor [10]. LVI has also been reported to be a strong predictor of recurrence or death for cancer patients in many studies, which is independent of lymph node metastasis. However, other studies have not confirmed the unfavorable prognostic effect of LVI in NSCLC. Up to date, LVI and BVI have not been recommended by the National Comprehensive Cancer Network to be decision factors in the TNM staging system, nor decision factors regarding adjuvant clinical treatment. Based on the discordant results obtained by a large number of studies on NSCLC, we performed a literature-based systematic review to better quantity the prognostic effects of LVI on the prognosis of the patients.

## Materials and Methods

### Publication Selection, Inclusion Criteria and Data Extraction

We searched the electronic databases PubMed (National Library of Medicine, Bethesda, USA) and EMBASE (Elsevier, Amsterdam, the Netherlands) between 1978 and 2012. Key words included non-small cell lung cancer, NSCLC, lymphatic vessel invasion, lymphatic involvement, lymphatic permeation, relapse, recurrence, prognostic, prognosis and outcome. The last search was updated in April 2012. The search was limited to English-language papers. This meta-analysis was limited to studies that dealt with the prognostic implications of LVI. The following criteria for eligibility among studies were set before selecting articles: (i) LVI was determined at least by hematoxylin and eosin (H&E) stain in surgically resected primary human lung tumors that had not received irradiation or chemotherapy prior to surgery, (ii) the relationship between LVI and survival was evaluated, and the results were published as a full paper, and (iii) available hazard ratio (HR) and 95% confidence interval (CI), or sufficient data are useful for examining HR and 95% CI. If a study detected lymphovascular invasion (including LVI or/and BVI) but did not analyze the association of LVI or BVI with survival separately, it will not be included in final meta-analysis.

The search and identification was independently conducted by three authors (J. Wang, Y. Guo and W. Zhao) according to a standardized approach, and the selection of a study was reached by discussion. Abstracts, reviews, other diseases and case reports were not included in this meta-analysis because of insufficient. When more than one of the same or overlapping publications was reported in several studies, only the most recently reported data or complete data were used for further combined analysis. We also performed a manual search from the references of relevant publications, including original articles and reviews, to identify additional records. For every study, last name of first author, year of publication, country of origin, patient resources, study size, methods for LVI evaluation, histology, and disease stage were collected. Three investigators (J. Wang, Y. Guo and W. Zhao) also independently performed methodological assessment. Disagreements were resolved by a third investigator (B. Wang). Quality scoring for each study was made according to the European Lung Cancer Working Party scale reported by Steels *et al* [4]. Studies included in the systematic review were denoted ‘eligible’, and those providing sufficient data for the meta-analysis are denoted ‘evaluable’.

### Statistical Methods

We performed separate meta-analyses using an adjusted or unadjusted hazard ratio (HR) for RFS and OS. In some studies, LVI was determined to be an independent prognostic indicator using multivariate analysis; HRs and 95% CIs were generally reported. Some studies reported the HR but did provide sufficient information on survival by LVI status; we thus calculated the HR and CIs according to the methods described by Parmar *et al* [11]. As shown in Table 1, the HR was calculated from the reported data by the total number of events, the log-rank statistic or its *P* value, or data from Kaplan-Meier survival curves. An observed HR >1 indicated a poor survival for the population with LVI. The χ^2^-based *Q* test was used to assess the heterogeneity of included studies [12]. A *P*-value <0.05 was considered to indicate significant heterogeneity. When the test of heterogeneity was significant, the random-effect model based on Mantel–Haenszel method would be used. A funnel plot and Egger’s linear regression test were used to investigate any possible publication bias [13]. The correlation between the score measurements was determined using the Spearman rank correlation coefficient. The score measurements involving the value of a discrete variable were calculated using the nonparametric Mann-Whitney U test. For all analyses, a two-sided *P* value of <0.05 was considered statistically significant. We read the Kaplan–Meier curves using Engauge Digitizer version 2.11 (free software downloaded from http://sourceforge.net). All analyses were performed using STATA version 11.0 software (Stata Corporation, College Station, TX, USA).

**Table 1:** Data source for the estimating of HR form included studies evaluating LVI and prognosis.

First author	Year	RFS		OS	
		Univariate	Multivariate analysis	Univariate	Multivariate analysis
Maeda *et al*.^39^	2012	N/A	N/A	*P*, event number	HR, 95%CI
Kawata *et al*.^24^	2012	HR, 95%CI	HR, 95%CI	N/A	N/A
Hanagiri *et al*.^40^	2011	N/A	N/A	*P*, event number	HR, 95%CI
Araki *et al*.^25^	2011	*P*, event number	HR, 95%CI	HR, 95%CI	N/A
Funai *et al*.^41^	2011	N/A	N/A	*P*, event number	HR, 95%CI
Sakai *et al*.^64^	2011	N/A	N/A	N/A	HR, 95%CI
Harada *et al*.^42^	2011	N/A	N/A	*P*, event number	HR, 95%CI
Ryuge *et al*.^43^	2011	N/A	N/A	HR, 95%CI	N/A
Maeda *et al*.^26^	2011	HR, 95%CI	HR, 95%CI	N/A	N/A
Maeda *et al*.^27^	2011	*P*, event number	HR, 95%CI	*P*, event number	HR, 95%CI
Maeda *et al*.^35^	2010	*P*, event number	HR, 95%CI	*P*, event number	HR, 95%CI
Yamaguchi *et al*.^44^	2010	N/A	N/A	N/A	HR, 95%CI
Shoji *et al*.^36^	2010	N/A	HR, 95%CI	N/A	N/A
Shimada *et al*.^45^	2010	N/A	N/A	*P*, event number	N/A
Kawachi *et al*.^37^	2009	N/A	HR, 95%CI	N/A	HR, 95%CI
Kawachi *et al*.^65^	2009	N/A	N/A	N/A	HR, 95%CI
Sun *et al*.^46^	2009	N/A	N/A	*P*, event number	HR, 95%CI
Hashizume *et al*.^47^	2009	N/A	N/A	*P*, event number	HR, 95%CI
Higashiyama *et al*.^49^	2009	N/A	N/A	HR, 95%CI	HR, 95%CI
Bodendorf *et al*.^50^	2009	N/A	N/A	*P*, event number	N/A
Mizuno *et al*.^48^	2008	N/A	N/A	*P*, event number	HR, 95%CI
Matsuguma *et al*.^68^	2008	N/A	N/A	N/A	HR, 95%CI
Cho *et al*.^28^	2008	*P*, event number	N/A	N/A	N/A
Hashizume *et al*.^38^	2008	N/A	HR, 95%CI	N/A	N/A
Saijo *et al*.^10^	2007	*P*, event number	HR, 95%CI	N/A	N/A
Shimizu *et al*.^51^	2005	N/A	N/A	*P*, event number	HR, 95%CI
Takanami *et al*.^52^	2005	N/A	N/A	*P*, event number	N/A
Takanami *et al*.^66^	2005	N/A	N/A	N/A	HR, 95%CI
Yamamoto *et al*.^33^	2004	*P*, event number	N/A	*P*, event number	N/A
Yoshida *et al*.^69^	2004	N/A	N/A	N/A	HR, 95%CI
Okada *et al*.^67^	2003	N/A	N/A	N/A	HR, 95%CI
Okada *et al*.^29^	2003	*P*, event number	N/A	*P*, event number	N/A
Poleri *et al*.^30^	2003	*P*, event number	HR, 95%CI	N/A	N/A
Maeshima *et al*.^53^	2002	*P*, event number	N/A	N/A	N/A
Saito *et al*.^54^	2002	N/A	N/A	*P*, event number	HR, 95%CI
Rigau *et al*.^34^	2002	HR, 95%CI	N/A	N/A	HR, 95%CI
Thomas *et al*.^59^	2002	N/A	N/A	*P*, event number	N/A
Suzuki *et al*.^70^	2002	N/A	N/A	N/A	HR, 95%CI
Moriya *et al*.^55^	2001	N/A	N/A	HR, 95%CI	HR, 95%CI
Yokose *et al*.^60^	2000	N/A	N/A	*P*, event number	N/A
Sukuki *et al*.^56^	1999	N/A	N/A	*P*, event number	N/A
Fu *et al*.^57^	1999	N/A	N/A	*P*, event number	N/A
Hirata *et al*.^61^	1998	N/A	N/A	N/A	HR, 95%CI
Bréchot *et al*.^31^	1996	*P*, event number	HR, 95%CI	*P*, event number	HR, 95%CI
Harpole *et al*.^62^	1995	N/A	N/A	*P*, event number	N/A
Fujisawa *et al*.^58^	1995	N/A	N/A	HR, 95%CI	HR, 95%CI
Ichinose *et al*.^63^	1995	N/A	N/A	*P*, event number	HR, 95%CI
Ogawa *et al*.^32^	1994	*P*, event number	HR, 95%CI	N/A	N/A

HR = hazard ratio; N/A = no available or no applicable; RFS = relapse-free survival; OS = overall survival.

## Results

### Study Selection and Characteristics

We perform an electronic data search in PubMed and EMBASE databases and yielded 154 citations. Additional 31 records were further identified via manually reviewing references. Five studies were excluded because an identical patient cohort occurred within another selected cohort [14]–[18]. Sixteen studies were not included in the overall meta-analysis because they investigated lymphovascular invasion and outcome in NSCLC patients (Table S1). The other excluded records include 2 reviews, 32 other diseases, 3 case reports, 16 non-English studies and 58 studies without available survival information (Table S1). Finally, 53 eligible studies published from 1992 to 2012 and satisfying the inclusion criteria for the systematic review and meta-analysis were identified. The PRISMA Checklist and Flow Diagram for the studies are shown in Checklist S1 and Figure S1, respectively.

The individual characteristics of the 53 eligible studies are summarized in Table S2. All included studies were reported retrospectively. A majority of studies included in this systematic review were based on Asian populations (79.2%), especially on Japanese people (73.6%). The total number of patients was 18,442 (range, 26–2295; median, 204). Overall, LVI appeared in 32.1% (median; range from 2.8% to 70.9%) of tumor samples. A total of 44 studies dealt with all types of NSCLC, 9 with adenocarcinoma alone. There were 22 studies reporting stage I patients and 2 studies without detailed stage information. The presence rate of LVI in stage I patients was 26.4% (median; range from 2.8% to 64.7%). In all included studies, formalin-fixed and paraffin-embedded resected specimens of NSCLC were collected retrospectively, and H&E-stained sections were reviewed. Tumor samples from nearly one-half of studies (49.1%) were investigated by H&E alone. Six studies (11.3%) investigated LVI by staining with H&E and D2-40 or LYVE-1 immunohistochemistry that is usually used as specific makers of lymphatic endothelium. Tumor specimens from 27 records were evaluated in combination with elastic staining (13 for elastica van Gieson, 13 for Victoria blue-van Gieson staining and 1 for elastica Masson staining) to distinguish between BVI and LVI. The published studies investigated multiple factors related to NSCLC outcome such as age, gender, smoking history, tumor size, histological differentiation, histological type, nodal status, LVI, BVI and pleural invasion. These clinicopathological variables including routine BVI and LVI were incorporated in most analyses (Table S3).

A total of 9.4% (5/53) of eligible publications [19]–[23] for systematic review were not evaluable owing to the lack of RFS or OS information even after writing to the authors for complementary information and remaining 48 studies are available for further meta-analysis. In univariate analysis for RFS, 10 eligible studies [10], [24]–[32] identified LVI as a poor prognostic factor for RFS and 2 identified LVI as not significant [33], [34]. However, two studies were not included in all meta-analyses because of overlap between cohorts [27], [35]. Nine studies reported significant RFS differences related to LVI status by multivariate analysis [24]–[26],[28],[30],[31],[36]–[38], and 3 reported no significant differences [10], [27], [32]. However, in the study by Cho *et al*., the significant risk for multivariate RFS was reported, but HR and 95% CI was not presented [28]. In addition, an overlap study was also excluded [27].

In univariate analysis for OS, 26 studies identified LVI as a significant prognostic factor [20], [22], [29], [31], [33], [34], [39]–[58], and 9 identified it as not significant for survival [19], [21], [25], [27], [59]–[63]. In multivariate analyses, 18 studies [20], [31], [37], [39], [41], [42], [47], [49], [51], [53], [55], [57], [58], [61], [64]–[67] were significant compared with 13 studies [19], [21], [27], [29], [34], [40], [44], [46], [48], [54], [63], [68], [69] with non-significant results. Of these included significant or non- significant studies, 3 have duplicated survival data [29], [37], [70] and one has uncompleted data [53].

Evaluability was not associated with positivity in the systematic review. The rate of significant results was 60.4% for evaluable trials (32/53) compared with 60.0% (3/5) for non-evaluable trials (*P* = 0.67) irrespective of whether these studies used univariate or multivariate analyses.

### Quality Assessment of Study

As shown in Table S4, the global quality assessment score, expressed as a percentage, ranged from 45.0% to 63.8% (median, 53.5%). There was no significant association between the global score and the number of patients in all eligible studies (Spearman *r* = 0.06; *P* = 0.65). As for the global score, no significant difference was found between the evaluable and the non-evaluable trials (*P* = 0.71). Similarly, no statistically significant difference was shown between the significant trials and the non-significant trials in univariate (*P* = 0.39) or multivariate analysis for OS (*P* = 0.49) (Table S4).

### Meta-analysis of the Effect of LVI on RFS for Overall Population

**Figure 1:**
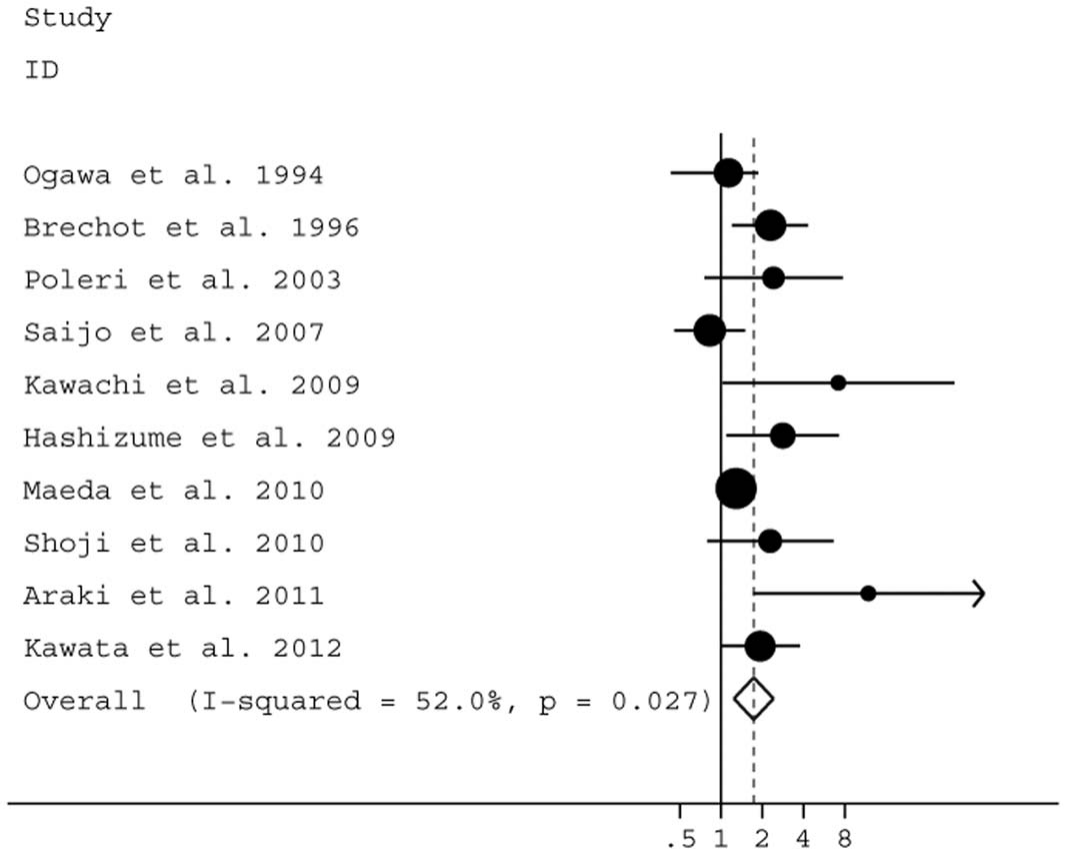
Forest plot showing the combined relative hazard ratio for relapse-free survival in all patient populations by multivariate analysis.

The results of meta-analysis of LVI and survival are presented in Table 2. In univariate analysis, LVI significantly increased the risk for cancer recurrence, with a combined HR of 2.48 (95% CI: 1.92–3.22; *P*<0.0001) (11 studies, 4,220 patients) [10], [24]–[26],[28]–[34]. There was evidence of significant inter-study heterogeneity (Q = 30.24; *I* ^2^ = 66.9%, *P* = 0.001). In multivariate analyses, patients with LVI were 1.73 times more likely to relapse compared with those without LVI (95% CI: 1.24–2.41; *P* = 0.001) (10 studies, 4,412 patients) [10], [24]–[26], [28], [30], [31], [36]–[38]. Significant heterogeneity occurred among these studies (Q = 18.77; *I* ^2^ = 52.0%, *P* = 0.027) (Fig. 1).

**Table 2:** Results of meta-analysis of LVI and survival in NSCLC patients.

Groups	Estimate of relative hazard				Homogeneity test		
	HR	95% CI	*P*		*Q* (df)	*I* ^2^ (%)	*P*
All studies							
Unadjusted RFS (11 studies, n = 4,220 )	2.48	1.92–3.22	<0.0001		30.24 (10)	66.9	0.001
Adjusted RFS (10 studies, n = 4,412)	1.73	1.24–2.41	0.001		18.77 (9)	52.0	0.027
Unadjusted OS (28 studies, n = 9,703)	1.97	1.75–2.23	<0.0001		62.17 (30)	51.7	<0.0001
Adjusted OS (25 studies, n = 9,423)	1.59	1.41–1.79	<0.0001		35.38 (24)	32.2	0.063
Studies using elastic stains with or without IHC							
Unadjusted RFS (5 studies, n = 3,272 )	2.26	1.74–2.94	<0.001		9.47 (4)	57.7	0.050
Adjusted RFS (6 studies, n = 3,583)	1.48	0.97–2.24	0.067		11.04 (5)	54.7	0.051
Unadjusted OS (18studies, n = 6,202)	1.82	1.62–2.06	<0.001		26.8 (17)	36.6	0.061
Adjusted OS (16 studies, n = 8,358)	1.47	1.29–1.67	<0.001		21.6 (15)	30.6	0.119
Studies using IHC							
Unadjusted RFS (2 studies, n = 584 )	2.69	0.99–7.30	0.053		2.50 (1)	59.9	0.114
Adjusted RFS (3 studies, n = 943)	2.37	0.63–8.84	0.200		2.50 (2)	79.5	0.008
Unadjusted OS (4 studies, n = 555)	2.22	1.66–2.96	<0.001		1.54 (3)	0	0.673
Adjusted OS (3 studies, n = 529)	2.22	1.01–5.11	0.047		3.95 (2)	49.4	0.139
I stage studies							
Unadjusted RFS (5 studies, n = 700)	2.31	1.84–2.91	<0.0001		2.85 (4)	0	0.584
Adjusted RFS (6 studies, n = 629)	1.91	1.14–3.17	0.013		9.25 (5)	45.9	0.100
Unadjusted OS (16 studies, n = 4,826 )	1.76	1.50–2.07	<0.0001		20.87 (15)	28.1	0.141
Adjusted OS (11 studies, n = 4,075)	1.70	1.38–2.10	<0.0001		18.59 (10)	40.8	0.069
AC studies							
Unadjusted RFS (2 studies, n = 375)	3.88	2.02–7.45	<0.0001		0 (1)	0	0.960
Adjusted RFS (2 studies, n = 679)	2.76	1.41–5.38	0.003		0 (1)	0	0.949
Unadjusted OS (4 studies, n = 647 )	3.44	2.08–5.70	<0.0001		5.95 (3)	49.6	0.114
Adjusted OS (4 studies, n = 553)	2.74	1.73–4.35	<0.0001		4.31 (3)	30.4	0.230

LVI = lymphatic vessel invasion; NSCLC = non-small-cell lung cancer; AC = adenocarcinoma; HR = hazard ratio; CI = confidence interval; OS = overall survival; RFS = relapse-free survival; IHC = immunohistochemistry.

### Meta-analysis of the Effect of LVI on OS for Overall Population

**Figure 2:**
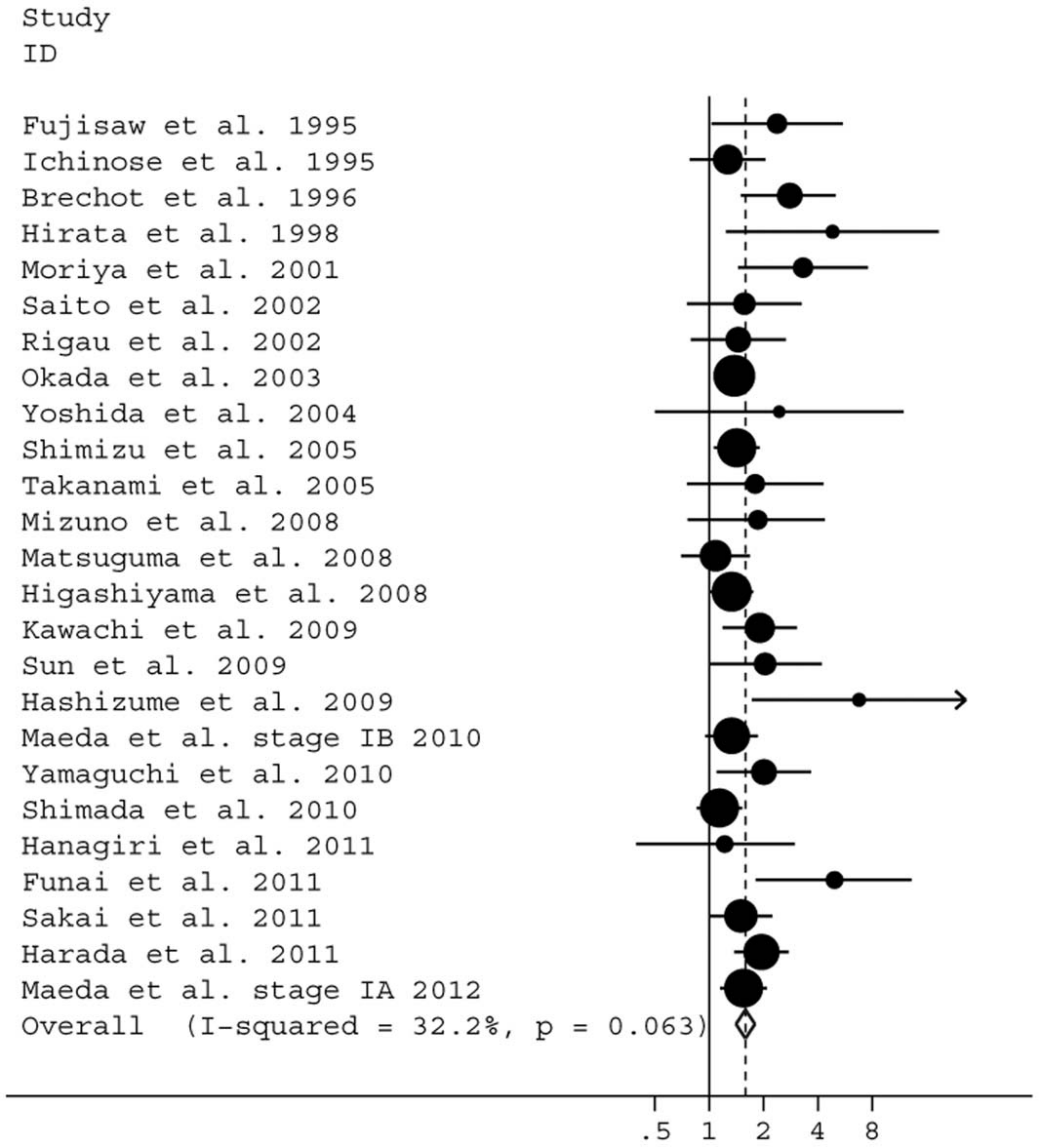
Forest plot showing the combined relative hazard ratio for overall survival in all patient populations by multivariate analysis.

We next analyze the association between LVI and OS in NSCLC patients by univariate (28 studies, comprising 9,703 cases) [25], [27], [31], [33], [34], [39]–[52], [54]–[60], [62], [63] or multivariate analysis (25 studies, comprising 9,423 cases) [27], [31], [34], [39]–[42], [44], [46]–[49], [51], [54], [57], [58], [61], [63]–[69]. The pooled HR estimate was 1.97 (95% CI: 1.75–2.21; *P*<0.0001) by univariate analysis with a significant heterogeneity (Q = 62.17; *I* ^2^ = 51.7%, *P*<0.0001). Our results also showed a risk was 59% higher for mortality (HR = 1.59; 95% CI: 1.41–1.79; *P*<0.0001) by multivariate analysis in LVI-positive patients compared with LVI-negative patients. Significant heterogeneity was not found among these studies (Q = 35.38; *I* ^2^ = 32.2%, *P* = 0.063) (Fig. 2).

### Meta-analysis of the Effect of LVI on Survival for Stage I or Adenocarcinoma Patients

**Figure 3:**
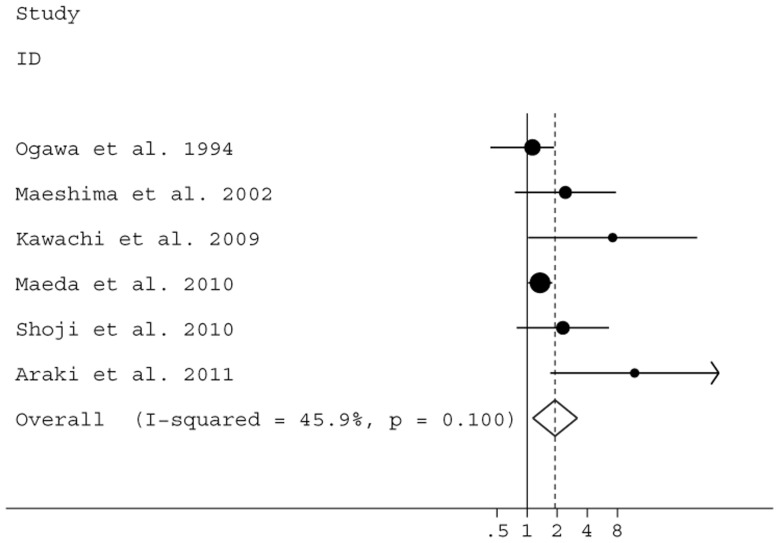
Forest plot showing the combined relative hazard ratio for relapse-free survival of stage I patients by multivariate analysis.

**Figure 4:**
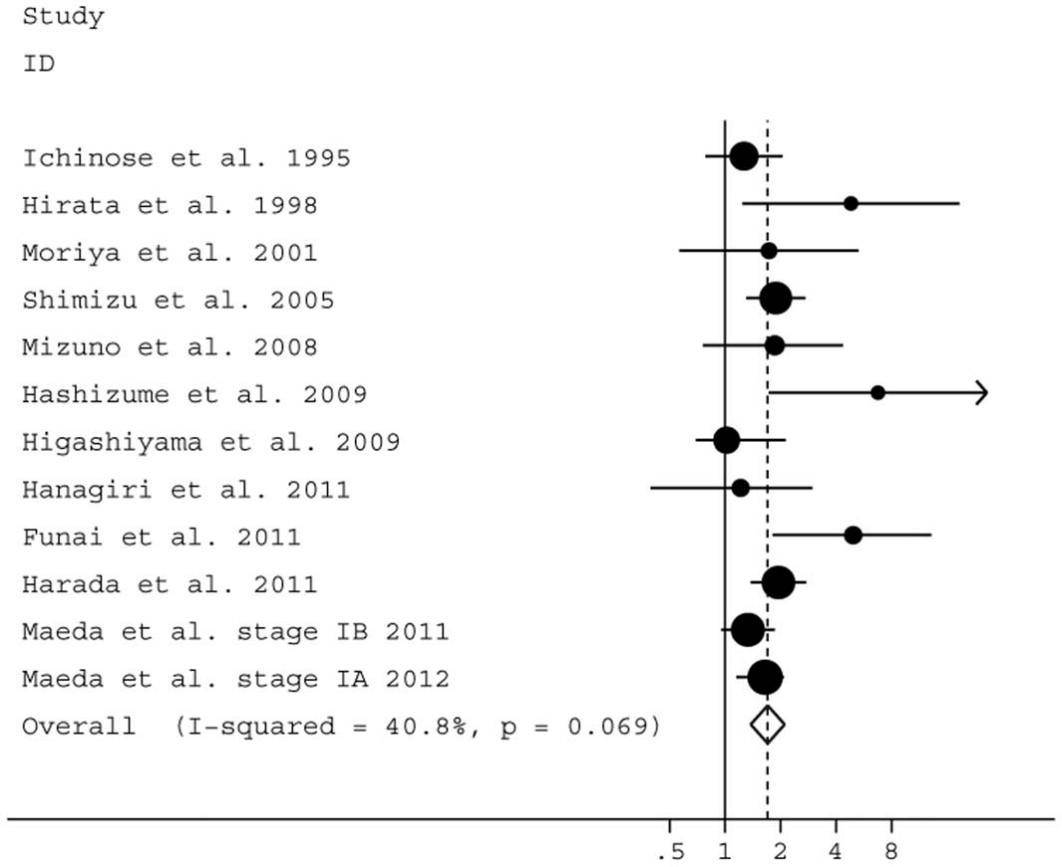
Forest plot showing the combined relative hazard ratio for overall survival of stage I patients by multivariate analysis.

We also reported the risk for recurrence and death in early-stage cancer patients with LVI. As shown in Table 2, using univariate and multivariate analysis, the summary HR estimates for RFS were 2.31 (95% CI: 1.84–2.91; *P*<0.0001) and 1.91 (95% CI: 1.14–2.91; *P* = 0.013), respectively (Fig. 3). Significant heterogeneity was not found. In the analysis for OS, LVI significantly appeared to increase the risk for mortality in stage I patients according to univariate (HR = 1.76, 95% CI: 1.50–2.07, *P*<0.0001) and multivariate analysis (HR = 1.70, 95% CI: 1.38–2.10, *P*<0.0001) (Fig. 4).

Considering only patients with adenocarcinoma, an increased risk for recurrence was observed using univariate (HR = 3.88, 95% CI: 2.02–7.45, *P*<0.0001) and multivariate (HR = 2.76, 95% CI: 1.41–5.38, *P = *0.003) analysis. We also found a significant higher unadjusted (HR = 3.44, 95% CI: 2.08–5.70, *P*<0.0001) and adjusted (HR = 2.74, 95% CI: 1.73–4.35, *P*<0.0001) risk for mortality of LVI-positive patients than that of LVI-negative patients. In these analyses, there was not still evidence of statistical heterogeneity (Table 2). These results suggest that LVI is a poor prognostic indicator and is independent of the tumor stage and histological type.

### Test of Heterogeneity and Subgroup Analyses

A high level of heterogeneity occurred when performing meta-analyses. Firstly, we conducted the subgroup analyses stratified by ethnicity or method for LVI evaluation. Although significant univariate risk for RFS in Asians and non-Asian populations was similar with the overall results, there was still evidence of statistical heterogeneity. Subgroup analyses by methods of LVI evaluation demonstrated that the combined HR for RFS by univariate analysis was 2.78 (95% CI: 1.57–4.91, *P*<0.001; heterogeneity test, *P* = 0.001) in studies evaluating LVI with H&E alone. By comparison, the combined HR for RFS by univariate analysis was 2.26 (95% CI: 1.74–2.94, *P*<0.001; heterogeneity test, *P* = 0.05) in studies evaluating LVI by elastic staining with or without immunohistochemistry, and 2.69 (95% CI: 0.99–7.30, *P* = 0.053; heterogeneity test, *P* = 0.114) in those evaluating LVI by immunohistochemistry alone. Furthermore analyses found that the report by Kawata *et al*. as a source of heterogeneity [24]. The heterogeneity disappeared when excluding this study and the value of pooled HR was not significantly altered (HR = 2.27; 95% CI: 1.84–2.80, *P*<0.001; heterogeneity test, Q = 11.98; *I* ^2^ = 33.2%, *P* = 0.152). However, we obtained a borderline multivariate HR for RFS (HR = 1.48; 95% CI: 0.97–2.24, *P = *0.067; heterogeneity test, Q = 11.04; *I* ^2^ = 54.7%, *P* = 0.051) in studies that utilized elastic staining or immunehistochemistry in defining LVI. Furthermore analysis showed the pooled multivariate HR estimate for RFS was 2.37 (95% CI: 0.63–8.84; *P = *0.200; heterogeneity test, Q = 2.5; *I* ^2^ = 79.5%, *P* = 0.008) in studies evaluating LVI by immunohistochemistry.

In univariate and multivariate analysis for OS in studies where LVI was investigated by elastic stains with or without immunohistochemistry, the summary HR estimates were 1.82 (95% CI: 1.62–2.06; *P*<0.0001; heterogeneity test, Q = 26.8; *I* ^2^ = 36.6%, *P* = 0.61) and 1.47 (95% CI: 1.29–1.67; *P*<0.001; heterogeneity test, Q = 21.6; *I* ^2^ = 30.6%, *P* = 0.119), respectively. When the meta-analysis was restricted into studies that utilized immunohistochemistry in defining LVI, the pooled HR estimate for OS was 2.22 (95% CI: 1.66–2.96; *P<*0.00001; heterogeneity test, *P* = 0.673) by univariate analysis and 2.22 (95% CI: 1.05–5.11; *P = *0.047; heterogeneity test, *P* = 0.139) by multivariate analysis. So subgroup analyses by methods of LVI evaluation did effectively decreased or removed the heterogeneity in univariate analysis for OS.

Publication bias statistics were determined using the methods of Egger *et al* [13]. No publication bias was found for the studies used for univariate analysis (*P* = 0.33) or for multivariate analysis of RFS (*P* = 0.14).

## Discussion

Microscopic metastasis begins with the local invasion by tumor cells into host stroma within or surrounding the primary tumor. When tumor cells penetrate a blood vessel or a peripheral lymphatic, they can detach, disseminate and arrest in the microvasculature through the circulation [71]. Micrometastases do not result from the random survival of cells released from the primary tumor but from the selective growth of specialized subpopulations of highly metastatic cells endowed with specific properties that enabled them to complete each step of the metastatic process. The cancer cells can spread to the lung and other sites through lymphatic vessel invasion and the regional lymph nodes, thoracic duct, superior vena cava, and pulmonary artery. The presence of vascular invasion by neoplastic cells indicates that the cancers are in a metastatic phase. Our previous meta-analysis found that LVI is a prognostic factor for survival in patients with NSCLC [8]. In the present study, we obtained summary statistics indicating that LVI status predicts poor survival in patients with NSCLC regardless of tumor size or lymph node status according to univariate or multivariate analysis. More importantly, LVI is an unfavorable prognostic determinant for patients with early stage disease or adenocarcinoma when adjusted for other prognostic factors.

LVI is significantly associated with lymph node metastasis and aggressive tumor behavior in NSCLC, indicating that it is a critical step in lymphogenous metastasis. The present analysis demonstrate that lymphatic invasion is present in 29.1% of overall patients and 26.1% of stage I patients. LVI is defined by the identification of tumor cells in the lumen of lymphatic vessels, which are often covered by endothelial cells and contained few lymphocytes. A pathological examination by H&E stain is helpful in recognizing LVI, but this method is usually impossible to distinguish between BVI and LVI, especially intratumoral areas. Lymphatic vessels do not contain elastic fibers, so they can not be confirmed by staining for elastic fibers which have been used as a routine pathological examination of BVI and pleural invasion. Elastic stains are less useful in excluding capillaries and are not useful in making the distinction between artifacts/stromal retraction and true lymphatic spaces. In fact, evaluation of LVI is relatively difficult using conventional H&E staining that showed a false-negative rate of 13.8 to18% and a false-positive rate of 4 to11.1% [72], [73]. As a result, studies reported the prognostic value of vessel invasion in tumor samples but did not differentiated between blood and lymphatic tumor emboli were not included in ultimate meta-analysis. This rate of LVI may have been underestimated because immunohistochemical methods were not performed in all studies. Although the monoclonal antibody D2-40 has often been used as a marker of lymphatic endothelium to identify tumor emboli in lymph vessels, it was recently found that D2-40 immunoreactivity was also detected in the basal cell layer of the squamous epithelium, stromal myofibroblasts, mesothelial cells, and lung cancer cells [74], [75]. In this meta-analysis, the combination of immunohistochemical staining with the lymph endothelium-specific marker D2-40 or LYVE-1 and H&E stain can indentify LVI and improve the accuracy of detecting LVI. Recently, Eynden *et al*. found that the combination of the lymph endothelium-specific marker D2-40 and the panendothelial marker CD34 might be of value in detecting and distinguishing between LVI and BVI in breast cancer specimens [76]. However, these special immunostaining markers are not used in routine pathological evaluation. In addition, the biggest difficulty appears be to detect lymphatic emboli and to distinguish them from possible tissue shrinkage. Unfortunately, in the present report only 6 studies investigated LVI with immunohistochemistry and a significantly increased risk for adjusted recurrence was not observed in studies investing LVI by immunohistochemistry. Based on our previous findings in identifying BVI as a significant prognostic factor [8], there could be potential differences between BVI and LVI in prediction of outcome for NSCLC patients and the effect of LVI on NSCLC prognosis does not seem to be more potent than that of BVI. The additional effect of the special immunostaining markers should be further validated in the future studies with a large patient population and standardization and accuracy of evaluating LVI and quality control is needed.

LVI can occur in intratumoral or extratumoral region. Hanagiri *et al*. found that lymphatic vessels and blood vessels are widely interconnected in the peritumorous region as spreading routes for the cancer cells [40]. Some studies also indicate that peritumoral lymphangiogensis and LVI are more common compared with the intratumoral lymphatics [77], and are present in significantly higher percentage of cases with lymph node metastasis, as compared to those without lymph node metastasis [78]. Tumors with LVI also showed a significantly higher rate of nodal metastases than those without LVI [79]. Experimental studies have showed that the functional lymphatics in the tumor margin alone are sufficient for lymphatic metastasis [80]. In this systematic review, however, only Saijo *et al*. analyzed intratumoral or extratumoral LVI separately and found that patients with extratumoral lymphatic invasion were more likely to relapse or develop a distant metastasis than those with intratumoral lymphatic invasion and without lymphatic invasion [10]. Shimada *et al*. reported intratumoral lymphatic permeation and extratumoral lymphatic permeation were found to be 152 and 92 cases, and the 5-year OS rates were 64.1% and 32.7%, respectively [45]. These results indicate that prognostic outcome of extratumoral lymphatic permeation is more unfavorable than that of intratumoral lymphatic permeation. Similar to BVI, almost all intratumoral lymphatic vessels are occluded by surrounding tumor cells and stromal cells, meaning that the intratumoral blood vessels and lymphatic vessels are not functional [42]. However, our meta-analysis focused on the effect of tumor LVI on the survival of NSCLC patients irrespective of whether these studies detected intratumoral or extratumoral LVI. To better understand the role of intratumoral or extratumoral LVI in lung cancer, further study is necessary.

Our meta-analyses had some limitations. The meta-analysis is based on retrospective data and the level of evidence is lower than that obtained by randomized controlled trials. However, similar attempts to examined prognostic influences of p53 expression, *RASSF1A* methylation and BVI status in patients with NSCLC yielded significant results. Data from published trials rather than individual patient data were used in the systematic review. In addition, in most of meta-analyses, there was evidence of significant heterogeneity although the random-effect model based on Mantel–Haenszel method rather than the fixed-effect model was applied. The wide heterogeneity in results could been associated with differences in some baseline characteristics of their designs, including population sample size, the duration of follow-up, the adjuvant treatment they might have received, year of publication, staining techniques, and different criteria for positive findings. For example, different methods for LVI evaluation such as H&E or combination with elastic-van Gieson or Victoria blue-van Gieson staining were used. In fact, LVI status ranged from 0 [81] to 70.9% [58]. According to previous report by Steels *et al.*, we used a methodology assessment on the treatment of lung cancer reported. However, this approach does not fully protect from potential bias because we could not take all the studies into account. These studies were finally maintained in the meta-analyses because the overall designs of studies were similar to those used in the other studies. TNM status remains the most important and differences in stage usually lead to heterogeneous results. However, heterogeneity was absent when the analysis was limited to studies of stage I or adenocarcinoma. Sensitive analysis showed individual studies contributed to significant heterogeneity. We concluded that heterogeneity probably come from differences of histological types and disease stages. Nonetheless, the precise reasons of heterogeneity remain unknown. Our results need to be substantiated by further prospective studies.

Usually, publication and reporting bias also has to be considered in meta-analyses. We did not look for unpublished papers, reviews or abstracts because the required data were usually available only in full publications. Positive results but not negative results tend to be accepted and published by journals. Another potential source of bias is related to the method used to extrapolate the HR. If the HR was not reported by author, it was calculated from the data included in the article or extrapolated from the survival curves, which involves making assumptions. In addition, each study adjusted for different covariates and only the studies that found significant results in univariate analysis performed multivariate analysis; thus, pooling the results may have produced bias. Nevertheless, no publication bias was detected using Egger’s test, suggesting that the summary statistics approximate the actual results.

In conclusion, our systematic and meta-analysis of the association between LVI and the risk of recurrence and death for NSCLC patients suggests that tumors with LVI, compared to those without LVI, may be significantly associated with a higher recurrence and mortality risk. In line with our previous report about the prognostic role of BVI, LVI appears to predictive of poor outcome among patients with NSCLC including early-stage diseases and aednocarcinomas. Based on the present findings, surgically treated NSCLC patients including stage I disease with LVI might benefit most from adjunct systematic chemotherapy. Vessel invasion, including LVI and BVI, might be useful in defining individual patients’ risk after radical surgery and should be incorporated in the new edition of the TNM classification. However, large, well-designed prospective studies with clinically relevant modeling and standard methodology to assess LVI are required in defining the management of patients with NSCLC.

## Supporting Information

Figure S1 **The flow of the included studies.**
(DOC)

Table S1 **Characteristics of literatures excluded in this systematic review.**
(DOC)

Table S2 **Data source for the estimating of HR form included studies evaluating lymphatic vessel invasion and outcome.**
(DOC)

Table S3 **Other clinicopathological variables in multivariate analysis of LVI and OS.**
(DOC)

Table S4 **Main characteristics and results of eligible studies evaluating LVI and RFS or OS in patients with NSCLC.**
(DOC)

Checklist S1 **PRISMA checklist.**
(DOC)
