# Supplementary material for: Clinical Significance and Role of Lymphatic Vessel Invasion as a Major Prognostic Implication in Non-Small Cell Lung Cancer: A Meta-Analysis
Source: PLoS One. 2012 Dec 20;7(12):e52704. doi: 10.1371/journal.pone.0052704 (PMC3527568; doi:10.1371/journal.pone.0052704)
Supplement: Figure S1 — The flow of the included studies. (DOC) [file pone.0052704.s001.doc]

**Figure S1.** PRISMA Flow Diagram

**Screening**

**Included**

**Eligibility**

**Identification**

Records identified through database searching
(n = 154)

Additional records identified through other sources
(n = 31)

Records after duplicates removed
(n = 5)

Records screened
(n = 180)

Records excluded
(n =127; 2 reviews, 32 other diseases, 3 case reports, 16 non-English articles, 16 studies investigating lymphovascular invasion and 58 studies without survival information)

Full-text articles assessed for eligibility
(n = 53)

Invaluable studies were excluded
(n = 5)

Studies included in qualitative synthesis
(n = 48)

Studies included in univariate meta-analysis for RFS
(n = 11)

Studies included in multivariate meta-analysis for RFS
(n = 10)

Studies included in univariate meta-analysis for OS
(n = 28)

Studies included in multivariate meta-analysis for OS
(n = 25)
