# Supplementary material for: Clinical Significance and Role of Lymphatic Vessel Invasion as a Major Prognostic Implication in Non-Small Cell Lung Cancer: A Meta-Analysis
Source: PLoS One. 2012 Dec 20;7(12):e52704. doi: 10.1371/journal.pone.0052704 (PMC3527568; doi:10.1371/journal.pone.0052704)
Supplement: Table S1 — Characteristics of literatures excluded in this systematic review. (DOC) [file pone.0052704.s002.doc]

**Table S1. Characteristics of literatures excluded in this systematic review**

| References | Year | Country | LVI evaluation | Reasons for exclusion |
| --- | --- | --- | --- | --- |
|
| 1 | 2011 | South Korea | No | Gastric cancer |
| 2 | 2011 | Japan | No | AC; case report |
| 3 | 2011 | France | Yes | NSCLC; lymphovascular invasion and survival |
| 4 | 2011 | China | No | NSCLC; survival analysis is not available |
| 5 | 2011 | USA | Yes | Breast cancer |
| 6 | 2011 | Poland | Yes | Esophageal cancer |
| 7 | 2011 | China | No | Pulmonary lymphangioleiomyomatosis |
| 8 | 2011 | China | No | Gastric cancer |
| 9 | 2011 | Japan | No | Breast cancer; case report |
| 10 | 2011 | South Korea | Yes | NSCLC; survival analysis is not available |
| 11 | 2011 | Japan | Yes | NSCLC; lymphovascular invasion and survival |
| 12 | 2011 | Japan | No | Breast cancer |
| 13 | 2011 | Japan | Yes | Endometrial carcinoma |
| 14 | 2011 | Japan | Yes | NSCLC; lymphovascular invasion and survival |
| 15 | 2011 | Japan | No | NSCLC; survival analysis is not available |
| 16 | 2011 | USA | Yes | Neuroendocrine carcinoma of the lung |
| 17 | 2010 | China | Yes | Extrapulmonary small cell carcinoma |
| 18 | 2010 | Turkey | No | NSCLC; survival analysis is not available |
| 19 | 2010 | South Korea | Yes | Sporadic colorectal cancer |
| 20 | 2010 | China | No | NSCLC; survival analysis is not available |
| 21 | 2010 | China | No | NSCLC; survival analysis is not available |
| 22 | 2010 | Japan | Yes | NSCLC; survival analysis is not available |
| 23 | 2010 | Switzerland | No | Review |
| 24 | 2010 | Japan | Yes | NSCLC; survival analysis is not available |
| 25 | 2010 | China | No | Esophageal small cell carcinoma |
| 26 | 2010 | China | Yes | NSCLC; lymphovascular invasion and survival |
| 27 | 2009 | Japan | Yes | NSCLC; survival analysis is not available |
| 28 | 2009 | Japan | No | NSCLC; survival analysis is not available |
| 29 | 2009 | Hungary | No | Small cell lung cancer |
| 30 | 2009 | USA | Yes | NSCLC; lymphovascular invasion and survival |
| 31 | 2008 | USA | No | Prostate cancer |
| 32 | 2008 | Germany | Yes | Breast cancer |
| 33 | 2008 | Kuwait | No | NSCLC; survival analysis is not available |
| 34 | 2008 | Japan | No | Hepatocellular carcinoma |
| 35 | 2008 | Japan | Yes | NSCLC; survival analysis is not available |
| 36 | 2008 | Belgium | Yes | NSCLC; lymphovascular invasion and survival |
| 37 | 2008 | Japan | Yes | Thyroid cancer |
| 38 | 2008 | China | Yes | NSCLC; survival analysis is not available |
| 39 | 2007 | Japan | Yes | Article in Japanese |
| 40 | 2007 | Japan | Yes | NSCLC; survival analysis is not available |
| 41 | 2007 | Japan | Yes | Article in Japanese |
| 42 | 2007 | Hungary | No | Article in Hungarian |
| 43 | 2007 | Japan | No | AC; survival analysis is not available |
| 44 | 2007 | Japan | Yes | AC; survival analysis is not available |
| 45 | 2007 | Japan | No | AC; survival analysis is not available |
| 46 | 2007 | Japan | No | AC; survival analysis is not available |
| 47 | 2005 | Japan | Yes | Hepatocellular carcinoma |
| 48 | 2005 | Finland | No | NSCLC; survival analysis is not available |
| 49 | 2005 | Japan | No | NSCLC; survival analysis is not available |
| 50 | 2005 | Austria | Yes | Neuroendocrine tumors of the lung |
| 51 | 2005 | Japan | Yes | NSCLC; survival analysis is not available |
| 52 | 2005 | Japan | Yes | Colorectal cancer |
| 53 | 2004 | Russia | Yes | Article in Russian |
| 54 | 2004 | Japan | No | NSCLC; survival analysis is not available |
| 55 | 2004 | Japan | No | AC; survival analysis is not available |
| 56 | 2004 | Japan | No | AC; survival analysis is not available |
| 57 | 2004 | Japan | No | AC; survival analysis is not available |
| 58 | 2004 | Japan | No | AC; survival analysis is not available |
| 59 | 2004 | Japan | No | Intrahepatic cholangiocarcinoma |
| 60 | 2003 | South Korea | No | Malignant pleural effusion |
| 61 | 2003 | Japan | Yes | AC; survival analysis is not available |
| 62 | 2003 | Japan | Yes | Ampullary carcinoma |
| 63 | 2003 | China | Yes | NSCLC; survival analysis is not available |
| 64 | 2003 | China | No | NSCLC; survival analysis is not available |
| 65 | 2003 | Japan | No | NSCLC; survival analysis is not available |
| 66 | 2003 | Japan | No | NSCLC; survival analysis is not available |
| 67 | 2002 | Japan | No | Article in Japanese |
| 68 | 2002 | Japan | Yes | AC; survival analysis is not available |
| 69 | 2001 | China | No | Thymoma |
| 70 | 2002 | Japan | Yes | AC; survival analysis is not available |
| 71 | 2001 | Germany | No | NSCLC; survival analysis is not available |
| 72 | 2001 | Japan | No | Article in Japanese |
| 73 | 2001 | Japan | Yes | NSCLC; lymphovascular invasion and survival |
| 74 | 2001 | Japan | Yes | NSCLC; survival analysis is not available |
| 75 | 2001 | Japan | Yes | Article in Japanese |
| 76 | 2001 | Japan | Yes | Gallbladder cancer |
| 77 | 2001 | Japan | No | NSCLC; survival analysis is not available |
| 78 | 2001 | Japan | No | NSCLC; survival analysis is not available |
| 79 | 2001 | USA | No | NSCLC; survival analysis is not available |
| 80 | 2000 | Japan | Yes | NSCLC; survival analysis is not available |
| 81 | 2000 | Spain | No | NSCLC; survival analysis is not available |
| 82 | 2000 | Japan | Yes | NSCLC; survival analysis is not available |
| 83 | 2000 | USA | No | AC; survival analysis is not available |
| 84 | 2000 | France | No | NSCLC; survival analysis is not available |
| 85 | 1999 | Japan | No | Case report |
| 86 | 1999 | Japan | Yes | NSCLC; survival analysis is not available |
| 87 | 1999 | USA | No | NSCLC; survival analysis is not available |
| 88 | 1999 | Italy | No | Non-Hodgkin's lymphoma |
| 89 | 1998 | Germany | Yes | Gastric cancer |
| 90 | 1997 | Greece | No | NSCLC; survival analysis is not available |
| 91 | 1998 | Japan | No | Pulmonary lymphoma. |
| 92 | 1997 | USA | No | papillary thyroid carcinoma |
| 93 | 1997 | USA | Yes | Colon cancer |
| 94 | 1997 | Japan | No | Cervical cancer |
| 95 | 1996 | Japan | No | Article in Japanese |
| 96 | 1996 | Germany | No | Article in German |
| 97 | 1996 | Japan | No | NSCLC; survival analysis is not available |
| 98 | 1995 | Italy | No | NSCLC; survival analysis is not available |
| 99 | 1995 | Canada | Yes | Exocrine pancreatic cancer |
| 100 | 1995 | France | Yes | NSCLC; lymphovascular invasion and survival |
| 101 | 1993 | Italy | No | NSCLC; survival analysis is not available |
| 102 | 1992 | Italy | No | NSCLC; survival analysis is not available |
| 103 | 1991 | Finland | No | NSCLC; survival analysis is not available |
| 104 | 1991 | Japan | Yes | Article in Japanese |
| 105 | 1991 | France | No | Article in French |
| 106 | 1990 | Japan | No | Review |
| 107 | 1991 | Japan | No | Article in Japanese |
| 108 | 1989 | Germany | No | NSCLC; survival analysis is not available |
| 109 | 1989 | Russia | No | Article in Russia |
| 110 | 1987 | Italy | No | NSCLC; survival analysis is not available |
| 111 | 1983 | Japan | No | Article in Japanese |
| 112 | 1983 | USA | Yes | NSCLC; survival analysis is not available |
| 113 | 1982 | Japan | No | NSCLC; survival analysis is not available |
| 114 | 1979 | Japan | No | NSCLC; survival analysis is not available |
| 115 | 1977 | Germany | No | Article in German |
| 116 | 2012 | Japan | No | Article in Japanese |
| 117 | 2011 | Japan | Yes | NSCLC; survival analysis is not available |
| 118 | 2001 | USA | Yes | NSCLC; lymphovascular invasion and survival |
| 119 | 2001 | Italy | Yes | NSCLC; lymphovascular invasion and survival |
| 120 | 2005 | France | Yes | NSCLC; lymphovascular invasion and survival |
| 121 | 2007 | Japan | Yes | NSCLC; lymphovascular invasion and survival |
| 122 | 2007 | Japan | Yes | NSCLC; survival analysis is not available |
| 123 | 2009 | Japan | Yes | NSCLC; lymphovascular invasion and survival |
| 124 | 2010 | China | Yes | NSCLC; survival analysis is not available |
| 125 | 2011 | Portugal | No | Papillary thyroid carcinoma |
| 126 | 2009 | China | Yes | NSCLC; lymphovascular invasion and survival |
| 127 | 2011 | Turkey | Yes | NSCLC; lymphovascular invasion and survival |

NSCLC = non-small cell lung cancer; SCLC = small cell lung cancer; AC = adenocarcinoma; SCC = squamous cell cancer; BAC = Bronchioloalveolar lung cancer; BVI = blood vessel invasion; UAE, United Arab Emirates.

**References**

1. Yoo YA, Kang MH, Lee HJ, Kim BH, Park JK, Kim HK, Kim JS, Oh SC. Sonic hedgehog pathway promotes metastasis and lymphangiogenesis via activation of Akt, EMT, and MMP-9 pathway in gastric cancer. *Cancer Res* 2011;71:7061-70.
2. Ohe M, Yokose T, Sakuma Y, Osanai S, Hasegawa C, Washimi K, Nawa K, Woo T, Hamanaka R, Nakayama H, Kameda Y, Yamada K, Isobe T. Stromal micropapillary pattern predominant lung adenocarcinoma--a report of two. *Diagn Pathol* 2011;6:92.
3. Massabeau C, Filleron T, Wakil G, Rouquette I, Bachaud JM, Leguellec S, Delisle MB, Toulas C, Mazieres J, Cohen-Jonathan Moyal E. The prognostic significance of lymphovascular invasion on biopsy specimens in lung cancer treated with definitive chemoradiotherapy. *Clin Lung Cancer* 2012;13:59-67.
4. Shao W, Wang W, Xiong XG, Cao C, Yan TD, Chen G, Chen H, Yin W, Liu J, Gu Y, Mo M, He J. Prognostic impact of MMP-2 and MMP-9 expression in pathologic stage IA non-small cell lung cancer. *J Surg Oncol* 2011;104:841-6.
5. Perentes JY, Kirkpatrick ND, Nagano S, Smith EY, Shaver CM, Sgroi D, Garkavtsev I, Munn LL, Jain RK, Boucher Y. Cancer cell-associated MT1-MMP promotes blood vessel invasion and distant metastasis in triple-negative mammary tumors. *Cancer Res* 2011;71:4527-38.
6. Kozłowski M, Naumnik W, Nikliński J, Milewski R, Lapuć G, Laudański J. Lymphatic vessel invasion detected by the endothelial lymphatic marker D2-40 (podoplanin) is predictive of regional lymph node status and an independent prognostic factor in patients with resected esophageal cancer. *Folia Histochem Cytobiol* 2011;49:90-7.
7. Gao J, Zhu P, Zhang S, Zhao S, Lu C, Chen H. A clinicopathological analysis of pulmonary lymphangioleiomyomatosis. *Zhongguo Fei Ai Za Zhi* 2011;14:378-82.
8. Gou HF, Chen XC, Zhu J, Jiang M, Yang Y, Cao D, Hou M. Expressions of COX-2 and VEGF-C in gastric cancer: correlations with lymphangiogenesis and prognostic implications. *J Exp Clin Cancer Res* 2011;30:14.
9. Hanada N, Tomiyama N, Hori K, Kusano S, Yoshida Y, Kawata K, Uchino R, Sakashita N. A case of recurrent breast cancer with lung metastasis resection showing four disease-free years under trastuzumab treatment. *Gan To Kagaku Ryoho* 2010;37:2905-7.
10. Min KH, Park SJ, Lee KS, Hwang SH, Kim SR, Moon H, Han HJ, Chung MJ, Lee YC. Clinical usefulness of D2-40 in non-small cell lung cancer. *Lung* 2011;189:57-63.
11. Shiono S, Abiko M, Sato T. Positron emission tomography/computed tomography and lymphovascular invasion predict recurrence in stage I lung cancers. *J Thorac Oncol* 2011;6:43-7.
12. Shibata MA, Ambati J, Shibata E, Albuquerque RJ, Morimoto J, Ito Y, Otsuki Y. The endogenous soluble VEGF receptor-2 isoform suppresses lymph node metastasis in a mouse immunocompetent mammary cancer model. *BMC Med* 2010;8:69.
13. Watanabe Y, Satou T, Nakai H, Etoh T, Dote K, Fujinami N, Hoshiai H. Evaluation of parametrial spread in endometrial carcinoma. *Obstet Gynecol* 2010;116:1027-34.
14. Igai H, Matsuura N, Tarumi S, Chang SS, Misaki N, Go T, Ishikawa S, Yokomise H. Clinicopathological study of p-T1aN0M0 non-small-cell lung cancer, as defined in the seventh edition of the TNM classification of malignant tumors. *Eur J Cardiothorac Surg* 2011;39:963-7.
15. Kitano H, Kageyama S, Hewitt SM, Hayashi R, Doki Y, Ozaki Y, Fujino S, Takikita M, Kubo H, Fukuoka J. Podoplanin expression in cancerous stroma induces lymphangiogenesis and predicts lymphatic spread and patient survival. *Arch Pathol Lab Med* 2010;134:1520-7.
16. Tsuta K, Raso MG, Kalhor N, Liu DD, Wistuba II, Moran CA. Histologic features of low- and intermediate-grade neuroendocrine carcinoma (typical and atypical carcinoid tumors) of the lung. *Lung Cancer* 2011;71:34-41.
17. Song Y, He J, Wu LY, Wang LH, Wang JW. Treatment and prognosis of extrapulmonary small cell carcinoma of 243 cases. *Zhonghua Zhong Liu Za Zhi* 2010;32:132-8.
18. Kiliçgün A, Turna A, Sayar A, Solak O, Urer N, Gürses A. Very important histopathological factors in patients with resected non-small cell: necrosis and perineural invasion. *Thorac Cardiovasc Surg* 2010;58:93-7.
19. Lim SB, Yu CS, Jang SJ, Kim TW, Kim JH, Kim JC. Prognostic significance of lymphovascular invasion in sporadic colorectal cancer. *Dis Colon Rectum* 2010;53:377-84.
20. Feng Y, Wang W, Hu J, Ma J, Zhang Y, Zhang J. Expression of VEGF-C and VEGF-D as significant markers for assessment of lymphangiogenesis and lymph node metastasis in non-small cell lung cancer. *Anat Rec* 2010;293:802-12.
21. Chen Z, Wang T, Luo H, Lai Y, Yang X, Li F, Lei Y, Su C, Zhang X, Lahn BT, Xiang AP. Expression of nestin in lymph node metastasis and lymphangiogenesis in non-small cell lung cancer patients. *Hum Pathol* 2010;41:737-44.
22. Kadota K, Huang CL, Liu D, Nakashima N, Yokomise H, Ueno M, Haba R. The clinical significance of the tumor cell D2-40 immunoreactivity in non-small cell lung cancer. *Lung Cancer* 2010;70:88-93.
23. Hillinger S, Weder W. Extended surgical resection in stage III non-small cell lung cancer. *Front Radiat Ther Oncol* 2010;42:115-21.
24. Zhou Q, Suzuki K, Anami Y, Oh S, Takamochi K. Clinicopathologic features in resected subcentimeter lung cancer--status of lymph node metastases. *Interact Cardiovasc Thorac Surg* 2010;10:53-7.
25. Song Y, Wang LH, He J, Wang JW. Treatment and prognosis of primary esophageal small cell carcinoma: a report of 151 cases. *Ai Zheng* 2009;28:303-7.
26. Li Z, Yu Y, Lu J, Luo Q, Wu C, Liao M, Zheng Y, Ai X, Gu L, Lu S. Analysis of the T descriptors and other prognosis factors in pathologic stage I non-small cell lung cancer in China. *J Thorac Oncol* 2009;4:702-9.
27. Maeda R, Isowa N, Onuma H, Miura H, Harada T, Touge H, Tokuyasu H, Kawasaki Y. The maximum standardized 18F-fluorodeoxyglucose uptake on positron emission tomography predicts lymph node metastasis and invasiveness in clinical stage IA non-small cell lung cancer. *Interact Cardiovasc Thorac Surg* 2009;9:79-82.
28. Iwakiri S, Nagai S, Katakura H, Takenaka K, Date H, Wada H, Tanaka F. D2-40-positive lymphatic vessel density is a poor prognostic factor in squamous cell carcinoma of the lung. *Ann Surg Oncol* 2009;16:1678-85.
29. Bogos K, Renyi-Vamos F, Dobos J, Kenessey I, Tovari J, Timar J, Strausz J, Ostoros G, Klepetko W, Ankersmit HJ, Lang G, Hoda MA, Nierlich P, Dome B. High VEGFR-3-positive circulating lymphatic/vascular endothelial progenitor cell level is associated with poor prognosis in human small cell lung cancer. *Clin Cancer Res* 2009;15:1741-6.
30. Varlotto JM, Recht A, Flickinger JC, Medford-Davis LN, Dyer AM, Decamp MM. Factors associated with local and distant recurrence and survival in patients with resected nonsmall cell lung cancer. *Cancer* 2009;115:1059-69.
31. Higashiyama M, Oda K, Okami J, Maeda J, Kodama K, Takenaka A, Nakayama T, Yoneda G.Prognostic value of intraoperative pleural lavage cytology for lung cancer without carcinomatous pleuritis: importance in patients with early stage disease during long-term follow-up. Prognostic value of intraoperative pleural lavage cytology for lung cancer. *Eur J Cardiothorac Surg* 2009;35:337-42.
32. Welter S, Jacobs J, Krbek T, Tötsch M, Stamatis G. Pulmonary metastases of breast cancer. When is resection indicated? *Eur J Cardiothorac Surg* 2008;34:1228-34.
33. Al-Sarraf N, Gately K, Lucey J, Aziz R, Doddakula K, Wilson L, McGovern E, Young V. Clinical implication and prognostic significance of standardised uptake value of primary non-small cell lung cancer on positron emission tomography: analysis of 176 cases. *Eur J Cardiothorac Surg* 2008;34:892-7.
34. Sugino T, Yamaguchi T, Hoshi N, Kusakabe T, Ogura G, Goodison S, Suzuki T. Sinusoidal tumor angiogenesis is a key component in hepatocellular carcinoma. *Clin Exp Metastasis* 2008;25:835-41.
35. Kadota K, Huang CL, Liu D, Ueno M, Kushida Y, Haba R, Yokomise H. The clinical significance of lymphangiogenesis and angiogenesis in non-small cell lung cancer patients. *Eur J Cancer* 2008;44:1057-67.
36. Poncelet AJ, Cornet J, Coulon C, Collard P, Noirhomme P, Weynand B; groupe d'oncologie thoracique des Cliniques Saint-Luc. Intra-tumoral vascular or perineural invasion as prognostic factors for long-term survival in early stage non-small cell lung carcinoma. *Eur J Cardiothorac Surg* 2008;33:799-804.
37. Naoi Y, Miyoshi Y, Taguchi T, Kim SJ, Arai T, Maruyama N, Tamaki Y, Noguchi S. Connexin26 expression is associated with aggressive phenotype in human papillary and follicular thyroid cancers.*Cancer Lett* 2008;262:248-56.
38. Wu N, Lv C, Yan S, Duan H, Zheng Q, Wang J, Xiong H, Yang Y. Systemic mediastinal lymph node dissection of right lung cancer: surgical quality control and analysis of mediastinal lymph node metastatic patterns. *Interact Cardiovasc Thorac Surg* 2008;7:240-3.
39. Kita H, Koshiishi Y, Masui K, Fujita A, Ootsuka K, Furuyashiki G, Nakazato Y, Takei H, Goya T. Risk factors of recurrence in resected stage I non-small cell lung cancer. *Kyobu Geka* 2007;60:883-7.
40. Aokage K, Ishii G, Nagai K, Kawai O, Naito Y, Hasebe T, Nishimura M, Yoshida J, Ochiai A. Intrapulmonary metastasis in resected pathologic stage IIIB non-small cell lung cancer: possible contribution of aerogenous metastasis to the favorable outcome. *J Thorac Cardiovasc Surg* 2007;134:386-91.
41. Ohta S, Hirose M, Ishibashi H, Muro H. Is adjuvant chemotherapy necessary for the peripherally located stage I? *Kyobu Geka* 2007;60:519-22.
42. Vörös A, Kaiser L, Somfay A, Pálinkás A. Primary lung cancer causing pulmonary artery microembolization and pulmonary hypertension. *Orv Hetil* 2007;148:1281-5.
43. Shio Y, Suzuki H, Kawaguchi T, Ohsugi J, Higuchi M, Fujiu K, Kanno R, Ohishi A, Gotoh M. Carbohydrate status detecting by PNA is changeable through cancer prognosis from primary to metastatic nodal site: A possible prognostic factor in patient with node-positive lung adenocarcinoma. *Lung Cancer* 2007;57:187-92.
44. Adachi Y, Nakamura H, Kitamura Y, Taniguchi Y, Araki K, Shomori K, Horie Y, Kurozawa Y, Ito H, Hayashi K. Lymphatic vessel density in pulmonary adenocarcinoma immunohistochemically evaluated with anti-podoplanin or anti-D2-40 antibody is correlated with lymphatic invasion or lymph node metastases. *Pathol Int* 2007;57:171-7.
45. Sakao Y, Miyamoto H, Sakuraba M, Oh T, Shiomi K, Sonobe S, Izumi H. Prognostic significance of a histologic subtype in small adenocarcinoma of the impact of nonbronchioloalveolar carcinoma components. *Ann Thorac Surg* 2007;83:209-14.
46. Ishiyama T, Kano J, Anami Y, Onuki T, Iijima T, Morisita Y, Yokota J, Noguchi M. OCIA domain containing 2 is highly expressed in adenocarcinoma mixed subtype with bronchioloalveolar carcinoma component and is associated with better prognosis. *Cancer Sci* 2007;98:50-7.
47. Natsuizaka M, Omura T, Akaike T, Kuwata Y, Yamazaki K, Sato T, Karino Y, Toyota J, Suga T, Asaka M. Clinical features of hepatocellular carcinoma with extrahepatic metastases. *J Gastroenterol Hepatol* 2005;20:1781-7.
48. He Y, Rajantie I, Pajusola K, Jeltsch M, Holopainen T, Yla-Herttuala S, Harding T, Jooss K, Takahashi T, Alitalo K. Vascular endothelial cell growth factor receptor 3-mediated activation of lymphatic endothelium is crucial for tumor cell entry and spread via lymphatic vessels. *Cancer Res* 2005;65:4739-46.
49. Ohno K, Utsumi T, Sasaki Y, Suzuki Y. Videopericardioscopy using endothoracic sonography for lung cancer staging. *Ann Thorac Surg* 2005;79:1780-2.
50. Schmid K, Birner P, Gravenhorst V, End A, Geleff S. Prognostic value of lymphatic and blood vessel invasion in neuroendocrine tumors. *Am J Surg Pathol* 2005;29:324-8.
51. Higashi K, Ito K, Hiramatsu Y, Ishikawa T, Sakuma T, Matsunari I, Kuga G, Miura K, Higuchi T, Tonami H, Yamamoto I. 18F-FDG uptake by primary tumor as a predictor of intratumoral lymphatic vessel invasion and lymph node involvement in non-small cell lung cancer: analysis of a multicenter study. *J Nucl Med* 2005;46:267-73.
52. Shiono S, Ishii G, Nagai K, Yoshida J, Nishimura M, Murata Y, Tsuta K, Nishiwaki Y, Kodama T, Ochiai A. Histopathologic prognostic factors in resected colorectal lung metastases. *Ann Thorac Surg* 2005;79:278-82.
53. Akopov AL, Dvorakovskaia IV. Vascular invasion by tumor in the absence of regional lymph node metastases in patients with locally advanced non-small cell lung cancer. *Vopr Onkol* 2004;50:417-20.
54. Suzuki K, Asamura H, Watanabe S, Tsuchiya R. Combined resection of superior vena cava for lung carcinoma: prognostic significance of patterns of superior vena cava invasion. *Ann Thorac Surg* 2004;78:1184-9.
55. Goto A, Niki T, Moriyama S, Funata N, Moriyama H, Nishimura Y, Tsuchida R, Kato JY, Fukayama M. Immunohistochemical study of Skp2 and Jab1, two key molecules in the degradation of P27, in lung adenocarcinoma. *Pathol Int* 2004;54:675-81.
56. Moriya Y, Iyoda A, Hiroshima K, Sekine Y, Shibuya K, Iizasa T, Saitoh Y, Fujisawa T. Clinicopathological analysis of clinical N0 peripheral lung cancers with a diameter of 1 cm or less. *Thorac Cardiovasc Surg* 2004;52:196-9.
57. Tsuta K, Ishii G, Yoh K, Nitadori J, Hasebe T, Nishiwaki Y, Endoh Y, Kodama T, Nagai K, Ochiai A. Primary lung carcinoma with signet-ring cell carcinoma components. *Am J Surg Pathol* 2004;28:868-74.
58. Tsuta K, Ishii G, Yoh K, Nitadori J, Hasebe T, Nishiwaki Y, Endoh Y, Kodama T, Nagai K, Ochiai A. Primary lung carcinoma with signet-ring cell carcinoma components: clinicopathological analysis of 39 cases. *Lung Cancer* 2004;44:43-51.
59. Sasaki A, Kawano K, Aramaki M, Ohno T, Tahara K, Kitano S. Correlation between tumor size and mode of spread in mass-forming intrahepatic cholangiocarcinoma. *Hepatogastroenterology* 2004;51:224-8.
60. Sayar A, Turna A, Solak O, Kiliçgün A, Urer N, Gürses A. Nonanatomic prognostic factors in resected nonsmall cell lung carcinoma: the importance of perineural invasion as a new prognostic marker. *Ann Thorac Surg* 2004;77:421-5.
61. Nomori H, Ohtsuka T, Naruke T, Suemasu K. Histogram analysis of computed tomography numbers of clinical T1N0M0 lung adenocarcinoma, with special reference to lymph node metastasis and tumor invasiveness. *J Thorac Cardiovasc Surg* 2003;126:1584-9.
62. Todoroki T, Koike N, Morishita Y, Kawamoto T, Ohkohchi N, Shoda J, Fukuda Y, Takahashi H. Patterns and predictors of failure after curative resections of carcinoma of the ampulla of Vater. *Ann Surg Oncol* 2003;10:1176-83.
63. Li Q, Dong X, Gu W, Qiu X, Wang E. Clinical significance of co-expression of VEGF-C and VEGFR-3 in non-small cell lung cancer. *Chin Med J* 2003;116:727-30.
64. Dong X, Qiu XS, Wang EH, Li QC, Gu W. Expression of vascular endothelial growth factor (VEGF) C and VEGF receptor 3 in non-small cell lung cancer. *Zhonghua Bing Li Xue Za Zhi* 2003;32:128-32.
65. Funai K, Yokose T, Ishii G, Araki K, Yoshida J, Nishimura M, Nagai K, Nishiwaki Y, Ochiai A. Clinicopathologic characteristics of peripheral squamous cell carcinoma of the lung. *Am J Surg Pathol* 2003;27:978-84.
66. Ishiwa N, Ogawa N, Shoji A, Maehara T, Hayashi Y, Takanashi Y, Yazawa T, Ito T. Correlation between lymph node micrometastasis and histologic classification of small lung adenocarcinomas, in considering the indication of limited surgery. *Lung Cancer* 2003;39:159-64.
67. Ota S, Muro H. The prognostic significance of lymphatic vessel and blood vessel invasion in peripherally located adenocarcinoma of the lung. *Nihon Rinsho* 2002;5:276-80.
68. Ikehara M, Oshita F, Kameda Y, Ito H, Ohgane N, Suzuki R, Saito H, Yamada K, Noda K, Mitsuda A. Expression of survivin correlated with vessel invasion is a marker of poor prognosis in small adenocarcinoma of the lung. O*ncol Rep* 2002;9:835-8.
69. Li J, Wang L, Zhang D. Cox multivariate analysis of prognosis and proposal on a modified staging system of thymoma. *Zhonghua Zhong Liu Za Zhi* 2001;23:500-2.
70. Ito H, Oshita F, Kameda Y, Suzuki R, Ikehara M, Arai H, Mitsuda A, Saito H, Yamada K, Noda K, Nakayama H. Expression of vascular endothelial growth factor and basic fibroblast growth factor in small adenocarcinomas. *Oncol Rep* 2002;9:119-23.
71. Passlick B, Sitar I, Sienel W, Thetter O, Morresi-Hauf A. Significance of lymphangiosis carcinomatosa at the bronchial resection margin in patients with non-small cell lung cancer. *Ann Thorac Surg* 2001;72:1160-4.
72. Yoneda S, Okabayashi K, Kawahara K, Iwazaki A, Yoshinaga Y, Yamamoto S, Ikeda K, Hamatake D, Takahashi M, Katoh F, Hayashi H, Yamaguchi R, Yoshida Y, Shirakusa T. Result of surgical treatment to early stage peripheral non-small cell lung cancer. *Kyobu Geka* 2001;54:932-7.
73. Aoki T, Tomoda Y, Watanabe H, Nakata H, Kasai T, Hashimoto H, Kodate M, Osaki T, Yasumoto K. Peripheral lung adenocarcinoma: correlation of thin-section CT findings with histologic prognostic factors and survival. *Radiology* 2001;220:803-9.
74. Kajita T, Ohta Y, Kimura K, Tamura M, Tanaka Y, Tsunezuka Y, Oda M, Sasaki T, Watanabe G. The expression of vascular endothelial growth factor C and its receptors in non-small cell lung cancer. *Br J Cancer* 2001;85:255-60.
75. Tanaka T, Kaneda Y, Fujita N, Ueda K, Saeki K, Sakano H, Matsuoka T, Sudo M, Hayashi M, Zempo N, Esato K. Assessment of stage IIB lung cancer from the pathological factors. *Kyobu Geka* 2001;54:359-62.
76. Wakai T, Shirai Y, Yokoyama N, Nagakura S, Watanabe H, Hatakeyama K. Early gallbladder carcinoma does not warrant radical resection. *Br J Surg* 2001;88:675-8.
77. Machida E, Nakayama J, Amano J, Fukuda M. Clinicopathological significance of core 2 beta1,6-N-acetylglucosaminyltransferase messenger RNA expressed in the pulmonary adenocarcinoma determined by in situ hybridization. *Cancer Res* 2001;61:2226-31.
78. Nakamura H, Kawasaki N, Hagiwara M, Ogata A, Kato H. Endoscopic evaluation of centrally located early squamous cell carcinoma of the lung. *Cancer* 2001;91:1142-7.
79. Skobe M, Hawighorst T, Jackson DG, Prevo R, Janes L, Velasco P, Riccardi L, Alitalo K, Claffey K, Detmar M. Induction of tumor lymphangiogenesis by VEGF-C promotes breast cancer metastasis. *Nat Med* 2001;7:192-8.
80. Ishida H, Sadahiro S, Suzuki T, Ishikawa K, Tajima T, Makuuchi H. c-erbB-2 protein expression and clinicopathologic features in colorectal cancer. *Oncol Rep* 2000;7:1229-33.
81. Medina JL. The value of transesophageal echography in the clinical staging of lung cancer. Ann Ital Chir 1999;70:847-9.
82. Hanagiri T, Kodate M, Nagashima A, Sugaya M, Dobashi K, Ono M, Yasumoto K. Bone metastasis after a resection of stage I and II primary lung cancer. *Lung Cancer* 2000;27:199-204.
83. Goldstein NS, Mani A, Chmielewski G, Welsh R, Pursel S. Immunohistochemically detected micrometastases in peribronchial and mediastinal. *Am J Surg Pathol* 2000;24:274-9.
84. Spaggiari L, Regnard JF, Magdeleinat P, Jauffret B, Puyo P, Levasseur P. Extended resections for bronchogenic carcinoma invading the superior vena cava system. *Ann Thorac Surg* 2000;69:233-6.
85. Sugio K, Sakada T, Saito G, Maruyama R, Nishioka K, Tominaga R, Nakanishi Y, Hara N, Sugimachi K Extended resection of the pulmonary artery and the aorta for primary lung cancer. Report of a case. *J Cardiovasc Surg* 1999;40:749-51.
86. Sato M, Ozeki Y, Aida S, Oshika Y, Deguchi H, Ono K, Haraguchi S, Ogata T, Tanaka S. Expression of alpha-smooth muscle actin in small bronchioloalveolar adenocarcinoma of the lung: assessment and comparison with noguchi criteria. *Oncol Rep* 1999;6:1217-21.
87. Bacha EA, Wright CD, Grillo HC, Wain JC, Moncure A, Keel SB, Donahue DM, Mathisen D Surgical treatment of primary pulmonary sarcomas. *Eur J Cardiothorac Surg* 1999;15:456-60.
88. Paulli M, Sträter J, Gianelli U, Rousset MT, Gambacorta M, Orlandi E, Klersy C, Lavabre-Bertrand T, Morra E, Manegold C, Lazzarino M, Magrini U, Möller P. Mediastinal B-cell lymphoma: a study of its histomorphologic spectrum based on 109 cases. *Hum Pathol* 1999;30:178-87.
89. Müller W, Schneiders A, Hommel G, Gabbert HE. Prognostic value of bcl-2 expression in gastric cancer. *Anticancer Res* 1998;18:4699-704.
90. Giatromanolaki A, Koukourakis MI, Theodossiou D, Barbatis K, O'Byrne K, Harris AL, Gatter KC. Comparative evaluation of angiogenesis assessment with anti-factor-VIII and. *Clin Cancer Res* 1997;3:2485-92.
91. Kawashima O, Hirai T, Kamiyoshihara M, Ishikawa S, Morishita Y. Early stage primary pulmonary lymphoma. *Oncol Rep* 1998;5:135-8.
92. Albores-Saavedra J, Housini I, Vuitch F, Snyder WH 3rd. Macrofollicular variant of papillary thyroid carcinoma with minor insular component. *Cancer* 1997;80:1110-6.
93. Takahashi Y, Tucker SL, Kitadai Y, Koura AN, Bucana CD, Cleary KR, Ellis LM. Vessel counts and expression of vascular endothelial growth factor as prognostic factors in node-negative colon cancer. *Arch Surg* 1997;132:541-6.
94. Katsuno S, Ishiyama T, Sakaguchi M, Takemae H. Carotid resection and reconstruction for advanced cervical cancer. *Laryngoscope* 1997;107:661-4.
95. Kawaguchi T. Adhesion molecules and carbohydrates in cancer metastasis. *Rinsho Byori* 1996;44:1138-46.
96. Becker HD. Endobronchial ultrasound--a new perspective in bronchology. *Ultraschall Med* 1996;17:106-12.
97. Okubo K, Yagi K, Yokomise H, Inui K, Wada H, Hitomi S. Extensive resection with selective cerebral perfusion for a lung cancer invading. *Eur J Cardiothorac Surg* 1996;10:389-91.
98. Fontanini G, Bigini D, Vignati S, Basolo F, Mussi A, Lucchi M, Chine S, Angeletti CA, Harris AL, Bevilacqua G. Microvessel count predicts metastatic disease and survival in non-small cell lung. *J Pathol* 1995;177:57-63.
99. Mao C, Domenico DR, Kim K, Hanson DJ, Howard JM. Observations on the developmental patterns and the consequences of pancreatic exocrine adenocarcinoma. Findings of 154 autopsies. Observations on the developmental patterns and the consequences of pancreatic. *Arch Surg* 1995;130:125-34.
100. Macchiarini P, Dulmet E, De Montpreville V, Chapelier A, Cerrina J, Le Roy Ladurie F, Dartevelle P. Prognostic significance of peritumoural blood and lymphatic vessel invasion by tumour cells in T4 non-small cell lung cancer following induction therapy. *Surg Oncol* 1995;4:91-9.
101. Macchiarini P, Fontanini G, Hardin MJ, Chuanchieh H, Bigini D, Vignati S, Pingitore R, Angeletti CA. Blood vessel invasion by tumor cells predicts recurrence in completely resected T1N0M0 non-small-cell lung cancer. *J Thorac Cardiovasc Surg* 1993;106:80-9.
102. Macchiarini P, Fontanini G, Hardin JM, Pingitore R, Angeletti CA. Most peripheral, node-negative, non-small-cell lung cancers have low. *J Thorac Cardiovasc Surg* 1992;104:892-9.
103. Lähde S, Päivänsalo M, Rainio P. Assessing non-resectability of lung cancer. The value of thoracic computed tomography. *Rofo* 1991;155:218-22.
104. Higashiyama M, Doi O, Kodama K, Tateishi R. Clinicopathological study of small adenocarcinoma of the lung. *Kyobu Geka* 1991;44:33-7.
105. Roeslin N, Warter A, Gasser B, Chakfe N, Weil G, Dumont P, Wihlm JM, Morand G, Witz JP. Non-aplastic N2 operated bronchial cancers. Multifactorial analysis of the prognosis. *Ann Chir* 1991;45:673-8.
106. Kuriyama K, Kadota T, Kuroda C. CT and MR imaging in the evaluation and staging of lung cancer. *Gan To Kagaku Ryoho* 1990;17:2140-7.
107. Hirayama T, Kaneda Y, Nawata S, Tanaka S, Esato K. Risk factors influencing lymph nodes metastasis in lung cancer with stage I, II or IIIA. *Nihon Kyobu Geka Gakkai Zasshi* 1990;38:2049-55.
108. Huwer H, Volkmer I, Hülsewede R, Hausinger F. A contribution on the prognostic significance of the tumor formula (pTNM) in squamous cell carcinoma of the bronchus. *Thorac Cardiovasc Surg* 1989;37:281-4.
109. Sakun FT, Kas'ianenko IV, Khrulenko LT, Babiĭ IaS, Lisitsa AM. Radiation and chemoradiation therapy of the lung following exploratory thoracotomy. *Klin Khir* 1989;5:8-10.
110. Rendina EA, Bognolo DA, Mineo TC, Gualdi GF, Caterino M, Di Biasi C, Facciolo F, Ricci C. Computed tomography for the evaluation of intrathoracic invasion by lung cancer. *J Thorac Cardiovasc Surg* 1987;94:57-63.
111. Shirakusa T. A clinicopathological study of metastasis of lung cancer and metastatic lung. *Nihon Geka Gakkai Zasshi* 1983;84:767-70.
112. Shields TW. Prognostic significance of parenchymal lymphatic vessel and blood vessel invasion in carcinoma of the lung. *Surg Gynecol Obstet* 1983;157:185-90.
113. Koga Y, Tomita M, Shibata K, Onitsuka T. Evaluation of limited resection of the lung for treatment of lung cancer. *Jpn J Surg* 1982;12:1-5.
114. Yoshida T, Shirakusa T, Shigematsu N, Ushijima Y, Inokuchi K. Histopathological factors predictive for prognosis of lung cancer. *Jpn J Surg* 1979;9:210-7.
115. Grunner A, Brunner W. The bronchial alveolar carcinoma as a solitary coin lesion. *Schweiz Med Wochenschr* 1977;107:211-2.
116. Tamari S, Nishikawa S, Aizawa R, Yamashina A, Motoyama H, Okita K, Chihara K. Clinical characteristics after surgery of non-small cell lung cancer which measures 20 mm or less in diameter. *Kyobu Geka* 2012;65:29-34.
117. Ishibashi T, Kaji M, Kato T, Ishikawa K, Kadoya M, Tamaki N. 18F-FDG uptake in primary lung cancer as a predictor of intratumoral vessel. *Ann Nucl Med* 2011;25:547-53.
118. Han H, Silverman JF, Santucci TS, Macherey RS, d'Amato TA, Tung MY, Weyant RJ, Landreneau RJ. Vascular endothelial growth factor expression in stage I non-small cell lung cancer correlates with neoangiogenesis and a poor prognosis. *Ann Surg Oncol* 2001;8:72-9.
119. Rena O, Oliaro A, Cavallo A, Filosso PL, Donati G, Di Marzio P, Maggi G, Ruffini E. Stage I non-small cell lung carcinoma: really an early stage? *Eur J Cardiothorac Surg* 2002;21:514-9.
120. Doddoli C, Aragon A, Barlesi F, Chetaille B, Robitail S, Giudicelli R, Fuentes P, Thomas P. Does the extent of lymph node dissection influence outcome in patients with stage I non-small-cell lung cancer? *Eur J Cardiothorac Surg* 2005;27:680-5.
121. Tsuchiya T, Akamine S, Muraoka M, Kamohara R, Tsuji K, Urabe S, Honda S, Yamasaki N. Stage IA non-small cell lung cancer: vessel invasion is a poor prognostic factor and a new target of adjuvant chemotherapy. *Lung Cancer* 2007;56:341-8.
122. Miyoshi K, Moriyama S, Kunitomo T, Nawa S. Prognostic impact of intratumoral vessel invasion in completely resected pathologic stage I non-small cell lung cancer. *J Thorac Cardiovasc Surg* 2009;137:429-34.
123. Zhou Q, Suzuki K, Anami Y, Oh S, Takamochi K. Clinicopathologic features in resected subcentimeter lung cancer--status of lymph node metastases. *Interact Cardiovasc Thorac Surg* 2010;10:53-7.
124. Lin Q, Li M, Shen ZY, Xiong LW, Pan XF, Gen JF, Bao GL, Sha HF, Feng JX, Ji CY, Chen M. Prognostic impact of vascular endothelial growth factor-A and E-cadherin expression in completely resected pathologic stage I non-small cell lung cancer. *Jpn J Clin Oncol* 2010;40:670-6.
125. Eloy C, Santos J, Soares P, Sobrinho-Simoes M. Intratumoural lymph vessel density is related to presence of lymph node metastases and separates encapsulated from infiltrative papillary thyroid carcinoma. *Virchows Arch* 2011;459:595-605.
126. Hsu CP, Hsia JY, Chang GC, Chuang CY, Shai SE, Yang SS, Lee MC, Kwan PC. Surgical-pathologic factors affect long-term outcomes in stage IB (pT2 N0 M0) non-small cell lung cancer: a heterogeneous disease. *J Thorac Cardiovasc Surg* 2009;138:426-33.
127. Yilmaz A, Duyar SS, Cakir E, Aydin E, Demirag F, Karakaya J, Yazici U, Erdogan Y. Clinical impact of visceral pleural, lymphovascular and perineural invasion in completely resected non-small cell lung cancer. *Eur J Cardiothorac Surg* 2011;40:664-70.
