# Supplementary material for: Clinical Significance and Role of Lymphatic Vessel Invasion as a Major Prognostic Implication in Non-Small Cell Lung Cancer: A Meta-Analysis
Source: PLoS One. 2012 Dec 20;7(12):e52704. doi: 10.1371/journal.pone.0052704 (PMC3527568; doi:10.1371/journal.pone.0052704)
Supplement: Table S2 — Data source for the estimating of HR form included studies evaluating lymphatic vessel invasion and outcome. (DOC) [file pone.0052704.s003.doc]

**Table S2.** Main characteristics and results of 53 eligible studies evaluating lymphatic vessel invasion and survival in patients with NSCLC

| First author | Year | Country | LVI/BVI evaluation | N | LVI | Stage | Histology | RFS |  | OS |  | Evaluable |
| --- | --- | --- | --- | --- | --- | --- | --- | --- | --- | --- | --- | --- |
|  |  |  |  |  | positive |  |  | Univariate | Multivariate analysis | Univariate | Multivariate analysis |  |
| *Maeda et al.*^39^ | 2012 | Japan | H&E, VVG | 1070 | 233 | IA | NSCLC | N/A | N/A | S | S | Yes |
| Kawata *et al*.^24^ | 2012 | Japan | H&E | 454 | 64 | I-IIIA | NSCLC | S | S | N/A | N/A | Yes |
| Hanagiri *et al*.^40^ | 2011 | Japan | H&E, EVG, D2-40 | 226 | 70 | I | NSCLC | N/A | N/A | S | NS | Yes |
| Araki *et al*.^25^ | 2011 | Japan | H&E, EVG, D2-40 | 26 | 7 | IB | NSCLC | S | S | NS | N/A | Yes |
| Funai *et al*.^41^ | 2011 | Japan | H&E, EVG | 229 | 32 | IA | NSCLC | N/A | N/A | S | S | Yes |
| Sakai *et al*.^64^ | 2011 | Japan | H&E, EVG | 548 | 265 | I-IV | NSCLC | N/A | N/A | N/A | S | Yes |
| Harada *et al*.^42^ | 2011 | Japan | H&E, EVG | 610 | 225 | I | NSCLC | N/A | N/A | S | S | Yes |
| Ryuge *et al*.^43^ | 2011 | Japan | H&E | 171 | 54 | I-III | NSCLC | N/A | N/A | S | N/A | Yes |
| Maeda *et al*.^27^ | 2011 | Japan | H&E, VVG | 320 | 23 | I | AC | S^a^ | S^a^ | N/A | N/A | Yes |
| Maeda *et al*.^35^ | 2011 | Japan | H&E, VVG | 434 | 134 | IB | NSCLC | S^a^ | NS^a^ | NS | NS | Yes |
| Maeda *et al*.^26^ | 2010 | Japan | H&E, VVG | 2295 | 700 | I-III | NSCLC | S | S | N/A | N/A | Yes |
|  |  |  |  | 1485 | 510 | I | NSCLC | S | S | N/A | N/A |  |
| Yamaguchi *et al*.^44^ | 2010 | Japan | H&E, VVG | 181 | 60 | I-IIIA | AC | N/A | N/A | S | NS | Yes |
| Shoji *et al*.^36^ | 2010 | Japan | H&E, EVG | 217 | 16 | IA | NSCLC | N/A | S | N/A | N/A | Yes |
| Shimada *et al*.^45^ | 2010 | Japan | H&E, VVG | 1000 | 244 | I-IV | NSCLC | N/A | N/A | S | N/A | Yes |
| Kawachi *et al*.^37^ | 2009 | Japan | H&E | 226 | 13 | I | NSCLC | N/A | S | N/A | S^b^ | Yes |
| Kawachi *et al*.^65^ | 2009 | Japan | H&E | 376 | 90 | I-IV | NSCLC | N/A | N/A | N/A | S | Yes |
| Sun *et al*.^46^ | 2009 | China | H&E, VVG, LYVE-1 | 82 | 46 | I-III | NSCLC | N/A | N/A | S | NS | Yes |
| Hashizume *et al*.^47^ | 2009 | Japan | H&E, EVG, D2-40 | 221 | 92 | IA | NSCLC | N/A | N/A | S | S | Yes |
| Higashiyama *et al*.^49^ | 2009 | Italy | H&E, VVG | 679 | 327 | I-IV | NSCLC | N/A | N/A | S | S | Yes |
|  |  |  |  | 393 | N/A | I | NSCLC | N/A | N/A | NS | NS |  |
| Bodendorf *et al*.^50^ | 2009 | Germany | H&E | 112 | 41 | IIB-III | NSCLC | N/A | N/A | S | N/A | Yes |
| Mizuno *et al*.^48^ | 2008 | Japan | H&E, VVG | 106 | 36 | IB | AC | N/A | N/A | S | NS | Yes |
| Cho *et al*.^28^ | 2008 | South Korea | H&E | 55 | 13 | I | AC | S | S | N/A | N/A | Yes |
| Matsuguma *et al*.^68^ | 2008 | Japan | H&E, EVG | 455 | 69 | I | NSCLC | N/A | N/A | N/A | NS | Yes |
| Hashizume *et al*.^38^ | 2008 | Japan | H&E, EVG, D2-40 | 359 | 41 | I-III | AC | N/A | S | N/A | N/A | Yes |
| Saijo *et al*.^10^ | 2007 | Japan | H&E, VVG, D2-40 | 558 | 94 | I-IIIA | NSCLC | S | NS | N/A | N/A | Yes |
| Shimizu *et al*.^51^ | 2005 | Japan | H&E, V**V**G | 1074 | 409 | I-III | NSCLC | N/A | N/A | S | S | Yes |
|  |  |  |  | 784 | N/A | I | NSCLC | N/A | N/A | S | S |  |
| Takanami *et al*.^52^ | 2005 | Japan | H&E | 134 | 77 | I-IIIA | NSCLC | N/A | N/A | S | N/A | Yes |
| Takanami *et al*.^66^ | 2005 | Japan | H&E | 79 | 43 | I-III | NSCLC | N/A | N/A | N/A | S | Yes |
| Yamamoto *et al*.^33^ | 2004 | Japan | H&E | 204 | 85 | I-III | NSCLC | NS | N/A | S | N/A | Yes |
| Yoshida *et al*.^69^ | 2004 | Japan | H&E | 79 | 42 | I-III | NSCLC | N/A | N/A | N/A | NS | Yes |
| Gabor *et al*.^19^ | 2004 | Austria | H&E | 72 | 22 | I-II | NSCLC | N/A | N/A | NS | NS | No |
| Sayar *et al*.^20^ | 2004 | Turkey | H&E | 82 | 37 | I-III | NSCLC | N/A | N/A | S | S | No |
| Okada *et al*.^23^ | 2003 | Japan | H&E | 136 | 31 | I-III | AC | N/A | N/A | S | N/A | No |
| Okada *et al*.^67^ | 2003 | Japan | H&E, EVG | 1000 | 321 | I-III | NSCLC | N/A | N/A | N/A | S | Yes |
| Okada *et al*.^29^ | 2003 | Japan | H&E, EVG | 265 | 54 | I-III | NSCLC | S | N/A | S | NS^c^ | Yes |
| Poleri *et al*.^30^ | 2003 | Argentina | H&E | 239 | 117 | I | NSCLC | S | S | N/A | N/A | Yes |
| Maeshima *et al*.^53^ | 2002 | Japan | H&E | 145 | 53 | I-IV | AC | N/A | N/A | S | S | Yes |
| Saito *et al*.^54^ | 2002 | Japan | H&E | 151 | 50 | I-III | NSCLC | N/A | N/A | S | NS | Yes |
| Rigau *et al*.^34^ | 2002 | France | H&E | 86 | 31 | I-IV | NSCLC | NS | N/A | S | NS | Yes |
| Thomas *et al*.^59^ | 2002 | France | H&E | 515 | 18 | I | NSCLC | N/A | N/A | NS | N/A | Yes |
| Suzuki *et al*.^70^ | 2002 | Japan | H&E, EMS | 164 | 76 | N/A | AC | N/A | N/A | N/A | S^d^ | Yes |
| Moriya *et al*.^55^ | 2001 | Japan | H&E | 102 | 42 | I-IV | NSCLC | N/A | N/A | S | S | Yes |
|  |  |  |  | 83 | 26 | I | AC | N/A | N/A | S | S |  |
| Yokose *et al*.^60^ | 2000 | Japan | H&E, EVG | 200 | 44 | I | AC | N/A | N/A | NS | N/A | Yes |
| Suzuki *et al*.^56^ | 1999 | Japan | H&E | 429 | 84 | I | NSCLC | N/A | N/A | S | N/A | Yes |
|  |  |  |  | 406 | 203 | II-IV |  | N/A | N/A | S | N/A |  |
| Fu *et al*.^57^ | 1999 | China | H&E | 158 | 112 | I-III | NSCLC | N/A | N/A | S | S | Yes |
| Hirata *et al*.^61^ | 1998 | Japan | H&E | 69 | 18 | I | NSCLC | N/A | N/A | NS | S | Yes |
| Duarte *et al*.^21^ | 1998 | USA | H&E | 104 | 21 | I | NSCLC | N/A | N/A | NS | NS | No |
| Bréchot *et al*.^31^ | 1996 | France | H&E | 96 | 57 | I-IV | NSCLC | S | S | S | S | Yes |
| Harpole *et al*.^62^ | 1995 | USA | H&E | 289 | 8 | I | NSCLC | N/A | N/A | NS | N/A | Yes |
| Fujisawa *et al*.^58^ | 1995 | Japan | H&E | 66 | 39 | I-III | NSCLC | N/A | N/A | S | S | Yes |
| Ichinose *et al*.^63^ | 1995 | Japan | H&E, VVG | 243 | 127 | I | NSCLC | N/A | N/A | NS | NS | Yes |
|  |  |  |  | 63 | 50 | II | NSCLC | N/A | N/A | NS | NS |  |
|  |  |  |  | 108 | 25 | IIIA | NSCLC | N/A | N/A | NS | NS |  |
| Ogawa *et al*.^32^ | 1994 | Japan | H&E, VVG | 128 | 57 | I | NSCLC | S | NS | N/A | N/A | Yes |
| Roberts *et al*.^22^ | 1992 | UK | H&E, EVG | 87 | 38 | N/A | NSCLC | N/A | N/A | S | N/A | No |

NSCLC = non-small cell lung cancer; AC = adenocarcinoma; N = overall cases; BVI (+) = cases for blood vessel invasion; S = significant relationship between BVI and relapse or survival; NS = no significant relationship between BVI and relapse or survival; N/A = no available or no applicable; RFS = relapse-free survival; OS = overall survival. H&E = hematoxylin and eosin stain; EVG = elastica van Gieson staining; VVG = victoria blue-van Gieson staining; EMS = elastica Masson staining. ^a^ Identical patient cohort occurred within another selected cohort (reference 26). ^b^ Identical patient cohort occurred within another selected cohort (reference 65). ^c^ Identical patient cohort occurred within another selected cohort (reference 67). ^d^ Identical patient cohort occurred within another selected cohort (reference 56).
