# Supplementary material for: Clinical Significance and Role of Lymphatic Vessel Invasion as a Major Prognostic Implication in Non-Small Cell Lung Cancer: A Meta-Analysis
Source: PLoS One. 2012 Dec 20;7(12):e52704. doi: 10.1371/journal.pone.0052704 (PMC3527568; doi:10.1371/journal.pone.0052704)
Supplement: Table S3 — Other clinicopathological variables in multivariate analysis of LVI and OS. (DOC) [file pone.0052704.s004.doc]

**Table S3.** Other clinicopathological variables in multivariate analysis of LVI and OS

| First author | Year | Other factors in multivariate analysis |
| --- | --- | --- |
| Maeda *et al*.^39^ | 2012 | Age, gender, smoking history, tumor size, tumor laterality, primary lobe, histological differentiation, BVI, pleural invasion, nodal status and intrapulmonary metastasis |
| Hanagiri *et al*.^40^ | 2011 | Gender, tumor size, histology, BVI and pleural invasion |
| Funai *et al*.^41^ | 2011 | Age, gender, surgical procedure, tumor size |
| Sakai *et al*.^64^ | 2011 | Age, gender, primary lobe, area of extension of lung cancer, pathologic T category, pathologic N category, pathologic M category, SHEATH, BVI and adjuvant therapy |
| Harada *et al*.^42^ | 2011 | Age, gender, histology, histological differentiation, tumor size, pleural invasion and BVI |
| Maeda *et al*.^26^ | 2010 | Age, gender, smoking history, FEV_1_, %, CEA, histology, histological differentiation, pleural invasion and BVI |
| Yamaguchi *et al*.^44^ | 2010 | Age, gender, tumor budding, smoking history, nodal status, pleural invasion and BVI |
| Kawachi *et al*.^37^ | 2009 | CEA level, pathological stage, pleural lavage cytology and BVI |
| Sun *et al*.^46^ | 2009 | Age, gender, histology, histological differentiation, pathologic T category, pathologic N category, BVI, LVD, pathologic stage, VEGF-C and Ki67, % |
| Hashizume *et al*.47 | 2009 | Age, gender, surgical procedure, pleural invasion, histology, tumor size and BVI |
| Mizuno *et al*.^48^ | 2008 | Age, gender, smoking history, CEA level, tumor size, histological differentiation, BVI, pleural invasion and BAC dominance |
| Matsuguma *et al*.^68^ | 2008 | Age, gender, smoking history, histology, tumor size, BVI and pleural invasion |
| Shimizu *et al*.^51^ | 2005 | Age, gender, histology, tumor size, tumor differentiation, pathological N status, pleural invasion and BVI, scar grade, nuclear atypia grade, mitotic index grade, CEA and surgical procedure |
| Takanami *et al*.^66^ | 2005 | Tumor size, nodal status, BVI, histology and Skp2 mRNA expression |
| Yoshida *et al*.^69^ | 2004 | Pathologic T category, pathologic N category, BVI, pathologic stage, cyclin B1 expression and Weel expression |
| Okada *et al*.^67^ | 2003 | Age, gender, CEA level, histology, pathologic stage, BVI and lavage cytolgy |
| Saito *et al*.^54^ | 2002 | Pathologic T category, pathologic N category, surgery procedure, curability, tumor size, pleural nvasion |
| Rigau *et al*.^34^ | 2002 | Age, gender, pathologic stage, BVI, Rb expression, Bcl2 expression and Ki67, % |
| Moriya *et al*.^55^ | 2001 | Gender, pathologic stage, nodal status, pleural invasion, BVI, fibroblastic resection and Laminin-5 expression |
| Fu *et al*.^57^ | 1999 | Age, gender, KPS, weight loss, pathologic T category, pathologic N category, histology, histological differentiation, BVI, index of positive oncoprotein stains and expression of proliferating cell nuclear antigen |
| Hirata *et al*.^61^ | 1998 | Histology, BVI, expression of proliferating cell nuclear antigen and CD44v6 |
| Bréchot *et al*.^31^ | 1996 | Tumor size, pathologic N category, pathologic stage, BVI, |
| Fujisaw *et al*.^58^ | 1995 | Age, gender, pathologic stage, BVI, pathologic T category, pathologic N category, intrapulmonary metastasis site |
| Ichinose *et al*.^63^ | 1995 | Tumor size, histological differentiation, pleural invasion and BVI |

OS, overall survival; LVI, lymphatic vessel invasion; BVI, blood vessel invasion; CEA, carcinoembryonic antigen
