# Supplementary material for: Clinical Significance and Role of Lymphatic Vessel Invasion as a Major Prognostic Implication in Non-Small Cell Lung Cancer: A Meta-Analysis
Source: PLoS One. 2012 Dec 20;7(12):e52704. doi: 10.1371/journal.pone.0052704 (PMC3527568; doi:10.1371/journal.pone.0052704)
Supplement: Table S4 — Main characteristics and results of eligible studies evaluating LVI and RFS or OS in patients with NSCLC. (DOC) [file pone.0052704.s005.doc]

**Table S4. Quality scores analysis of the 53 eligible studies by the European Lung Cancer Working Party score according to studies characteristics**

| Studies (*n*) | Design (/10) | Laboratory method (/10) | Generalizability (/10) | Results analysis | Global score (%) |
| --- | --- | --- | --- | --- | --- |
| All (53) | 4.4 | 5.1 | 5.9 | 6.3 | 54.3 |
| Patients number |  |  |  |  |  |
| Spearman *r* | 0.01 | 0.01 | 003 | 0.10 | 0.06 |
| *P* | 0.93 | 0.99 | 0.83 | 0.50 | 0.65 |
| Evaluable for meta-analysis |  |  |  |  |  |
| Yes (48) | 4.4 | 5.1 | 5.9 | 6.3 | 54.2 |
| No (5) | 4.8 | 5.3 | 5.8 | 6.2 | 55.2 |
| *P* | 0.27 | 0.50 | 0.70 | 0 .88 | 0.71 |
| Significant results for OS in univariate analysis |  |  |  |  |  |
| Yes (26) | 4.4 | 5.2 | 5.9 | 6.3 | 54.6 |
| No (9) | 4.0 | 5.6 | 6.4 | 7.5 | 58.8 |
| *P* | 0.48 | 0.37 | 0.37 | 0.16 | 0.39 |
| Significant results for OS in multivariate analysis |  |  |  |  |  |
| Yes (18) | 4.5 | 5.3 | 6.0 | 6.5 | 55.7 |
| No (13) | 4.0 | 5.6 | 6.4 | 7.5 | 58.8 |
| *P* | 0.45 | 0.45 | 0.46 | 0.22 | 0.49 |

Scores distributions are summarized by median values. OS = overall survival.
